# *Juniperus phoenicea* extract-loaded nanostructured lipid carriers for potent antimicrobial and anticancer combination therapy

**DOI:** 10.1186/s13568-026-02054-0

**Published:** 2026-09-04

**Authors:** Passant A. Ismail, Bassma H. Elwakil, Mohamed Hagar, Zakia A. Olama

**Affiliations:** 1 Botany & Microbiology Department, Faculty of Science, Alexandria University, Alexandria, 21500 Egypt; 2 Medical Laboratory Technology Program, Faculty of Applied Health Sciences Technology, Galala University, Suez, Egypt; 3 Chemistry Department, Faculty of Science, Alexandria University, Alexandria, 21500 Egypt

**Keywords:** *Juniperus phoenicea* L., Nanostructured lipid nanoparticle, Network pharmacology-based analysis, Cytotoxicity, Antibacterial, Anticancer

## Abstract

This study evaluates the antimicrobial properties of *Juniperus phoenicea* L. against multidrug-resistant microbes, highlighting potential synergistic effects of combining the *J. phoenicea* L. newly synthesized nanoparticles with conventional antibiotics and anticancer agents. *Juniperus phoenicea* L. ethanolic and ethyl acetate extracts were prepared. Antibacterial activity against a panel of multidrug-resistant microbes showed the ethanolic extract as superior to the ethyl acetate extract, with *Acinetobacter baumannii* being the most susceptible organism (minimum inhibitory MIC = 12.5 µg/mL). Phytochemical profiling of the ethanolic extract revealed predominant components, namely: Apigenin-7-glucoside, α-pinene, and Cedrol. Network pharmacology-based analysis suggested the potent effect of these compounds as anticancer and antibacterial agents. *J. phoenicea* L. nanoparticles were synthesized by a combined hot-melt dispersion and homogenization approach. Characterization yielded a zeta vesicle size of 98.6 nm, PDI 0.23, zeta potential + 48.5 mV, EE% 92.4%, and TEM size 76.2 nm. The nanoparticles markedly enhanced antibacterial activity (MIC = 6.25 µg/mL) with complete eradication of *A. baumannii* after 16 h. The combination with Amoxicillin and Cefoxitin showed synergistic effects. In anticancer assays, nanoparticles outperformed Fluorouracil, with IC_50_ = 9.05 ± 0.49 µg/mL (≈ 8.7-fold higher anticancer potency than the crude extract). The Fluorouracil–nanoparticle combination further reduced IC_50_ to 6.12 ± 0.41 µg/mL. Hence, it was concluded that *J. phoenicea* L. ethanolic extract exhibits potent antibacterial and anticancer properties and provides an effective nanoparticle precursor. Synergistic interactions with antibiotics and Fluorouracil suggest a multifaceted therapeutic strategy against infections and cancer, meriting further in vivo studies and mechanistic exploration.

**Supplementary Information:**

The online version contains supplementary material available at https://doi.org/10.1186/s13568-026-02054-0.

## Introduction

For millennia, Medicinal plants have been used as cures for human illnesses thanks to their chemical components that possess therapeutic properties (Izah et al. 2023). Most of the world’s population was found to rely primarily on traditional medicines to treat various health conditions (Che et al. 2023). In general, wild medicinal plants contain abundant flavonoids, which have been recognized as advantageous elements with antimicrobial and anticancer effects. *Juniperus* spp is a prominent member of the Cupressaceae family, with around sixty-seven different species that are widely distributed across the globe. It is characterized by its needle-like juvenile leaves and scale-like adult leaves. The fruits, which are referred to as berries, have been utilised as a culinary seasoning in European cuisine, as a contraceptive method, as well as a way of treating Diabetes by Native Americans. *J. phoenicea* berries were also discovered in ancient Egyptian tombs and were found to naturally grow in Egypt’s Sinai region and the wider Mediterranean area (Al Masoudi et al. 2023). An analysis of the leaves and berries of the plant revealed a high concentration of essential oils, carbohydrates, glycosides, sterols, triterpenes, flavonoids, phenolic compounds, tocopherols, and ascorbic acid (Mansour et al. 2023). Studies demonstrated a potent antimicrobial activity for *J. phoenicea* against various bacterial strains, as well as cytotoxic effect on breast and colon cancer cell lines (Al Masoudi et al. 2023). The powdered leaves have been used to treat bronchial infections and promote urine production. In other countries, *Junipers* spp. are also used as traditional medicines to treat respiratory illnesses and skin conditions (Öztürk et al. 2011), as well as rheumatic disorders and gall bladder stones (Šarić-Kundalić et al. 2011).

The use of natural sources namely extracts from wild medicinal plants, to produce nanoparticles (i.e. green synthesis) is an innovative method that could potentially have an impact on the management of various illnesses and cancers. This is referred to as Phyto-Nanomedicine (Elbhnsawi et al. 2023). Green synthesis nanoparticles provide an environmentally friendly, cost-effective, and renewable platform for different therapeutic and diagnostic applications of nanoparticles (Rajkumari et al. 2019). Lipid-based nanoparticles (LNPs) have ascended as flexible nanoscale vehicles for biomedical applications, owing to their biodegradability, biocompatibility, and capability to cradle diverse therapeutic cargoes—traits that favor clinical translation (Ganesan et al. 2017). Within the LNP family, architectures such as solid lipid nanoparticles (SLNs), liposomes, nanostructured lipid carriers (NLCs), and micelles are well established. Among them, NLCs—defined by a heterogeneous blend of liquid and solid lipids—offer superior loading efficiency, enhanced physical stability, and more controllable release compared with SLNs (Viegas et al. 2023).

Synthesizing *J. phoenicea* in nanoform would enhance its biological activity and broaden its potential applications, especially in medicine. To the best of our knowledge, there is a study gap in formulating *J. phoenicea* extract-loaded nanoparticles (NLC, SLN, or chitosan-based). Hence, in our study, we successfully synthesized novel nanoparticles from *J. phoenicea* leaf extract-nanostructured lipid carrier (JP-NLCs). The characterization, antibacterial effect against multidrug-resistant organisms, and anticancer properties were assessed. This investigation signifies a promising new direction in harnessing the potential of wild medicinal plants in their nanoform as effective remedies.

## Materials and methods

### Materials

#### Microorganisms

Bacterial isolates of *Acinetobacter baumannii*, *Enterobacter aerogenes*, methicillin-resistant *Staphylococcus aureus* (MRSA), *Escherichia coli*, and *Proteus mirabilis* were employed in this investigation. The tested isolates were supplied and authenticated by the Microbiology Department at Al-Shatby Pediatric Hospital, Alexandria, using the VITEK system (BIOMERIEUX, USA). The *A. baumannii* strain was added to the World Data Centre for Microorganisms Culture Collection at Ain Shams University (CCASU) (isolate number CCASU-P79). *E. coli* 5922 and MRSA 13,300 ATCC strains were kindly provided by the U.S Naval Medical Research Unit (NMRU) in Cairo. All strains were preserved in brain–heart infusion glycerol broth and routinely subcultured monthly onto fresh media to maintain viability.

### Cancer cells

The A-549 human lung carcinoma cell line was sourced from the American Type Culture Collection (ATCC, Rockville, MD). Reagents spanning MTT (3-[4,5-dimethylthiazol-2-yl]-2,5 diphenyl tetrazolium bromide), Dimethyl sulfoxide (DMSO), and trypan blue dye were procured from Sigma-Aldrich (St. Louis, MO, USA). Additional culture components—Fetal Bovine Serum, HEPES buffer, RPMI-1640, gentamicin, L-glutamine, and 0.25% Trypsin-EDTA—were acquired from Lonza (Belgium).

#### ***Juniperus phoenicea*** L. sample collection

Juvenile leaves of *J. phoenicea* L. were harvested in March 2023 from Antoniades Garden, Alexandria, Egypt. Plant identity was confirmed by botanists and teaching staff in the Faculty of Science at Alexandria University. The voucher specimen (JP 11) has been deposited in the herbarium of the Department of Pharmacognosy, Faculty of Pharmacy, Alexandria University, serving as the reference material for this study. Prior to phytochemical analysis, samples were air-dried at ambient temperature.

The collection and publication of plant material were authorized by the Institutional Animal Care and Use Committees (IACUCs) of the Faculty of Science, Alexandria University, under approval number AU04-206-31-10-2023.

## Methods

### Preparation of crude bioactive extracts of ***Juniperus phoenicea***L.

Dried leaves (25 g) were subjected to maceration with 95% ethanol and then ethyl acetate (80:20 v/v), performed for 24 h at ambient temperature (25 °C) (Draoui et al. 2020). Filtration followed by concentration was accomplished using a rotary evaporator under reduced pressure at 45 °C to yield the respective extracts (the final yield was 5.40 g, corresponding to 21.6%). Collection of *J. phoenicea* L. material was conducted in full compliance with applicable institutional, national, and international guidelines and regulations.

## Antimicrobial activities of the rich polyphenol extract

The antimicrobial potential of the polyphenol-rich extract was evaluated using the disk diffusion method and broth microdilution following 2017 CLSI guidelines. Inocula were standardized to a 0.5 McFarland turbidity standard (approx. 1.5 × 10^8^ CFU/mL) and further diluted to a final test concentration of 5 × 10^5^ CFU/mL. Assays were conducted in Mueller-Hinton Broth (MHB) and incubated at 37 °C for 24 h in triplicate. The Minimum Inhibitory Concentration (MIC) was defined as the lowest concentration of extracts showing no visible growth. The Minimum Bactericidal Concentration (MBC) was determined by subculturing 10 µL from non-turbid wells onto Mueller-Hinton Agar (MHA); the MBC was defined as the lowest concentration resulting in a reduction of the initial inoculum (no colony growth). Microbial lethality was further characterized via time-kill curves by monitoring log_10_ CFU/mL reductions at specific MIC multiples over a 24-hrs period (Abbey et al., 2019).

### ***Juniperus phoenicea*** L. bioactive extract analyses

#### Measurements of the total polyphenolic contents

Total phenolics were quantified by the Folin–Ciocalteu assay, which detects phenolic-induced blue complex in alkaline medium (Taga et al. 1984). A gallic acid standard curve was prepared from 0.01 to 0.05 mg/mL in five incremental steps (0.01 mg/mL each), with reaction tubes: sample, gallic acid standards, and a blank. Each tube received 1 mL test solution (or standard), followed by 2.5 mL of sodium bicarbonate to establish the alkaline medium. After 2 h at 25 °C, absorbance was recorded at 765 nm using an Optima spectrophotometer and interpolated from the gallic acid standard curve. The blank served to baseline the instrument prior to each reading, and the assay was performed in triplicate. Phenolic concentration (mg/mL) was calculated from the standard curve as: Phenolic concentration = [(AE − AB) − I] / S, where AE is the mean sample absorbance, AB is the mean blank absorbance, I is the intercept, and S is the slope.

### Measurements of flavonoid content

Flavonoid content was quantified by the aluminum chloride colorimetric assay, relying on the formation of Al-flavonoid complexes that yield a measurable absorbance at 510 nm (Zhishen et al. 1999). A quercetin standard curve was generated from 0.1 to 0.5 mg/mL. In each assay, 0.25 mL of the sample (1 mg/mL), quercetin standards, or distilled water (blank), was combined with 0.75 mL of 5% NaNO_2_ and 1.25 mL of distilled water, then incubated for 5 min at room temperature. Subsequently, 0.15 mL of AlCl_3_ was added, followed by 2 mL of NaOH and thorough mixing. Absorbance was measured at 510 nm with an Optima spectrophotometer, with the assay performed in triplicate. Flavonoid concentration (mg/mL) was calculated as: Flavonoid concentration = [(AE − AB) − I] / S, where AE is the mean sample absorbance, AB the mean blank absorbance, I the intercept, and S the slope of the standard curve.

### Measurements of tannin content

Peterson et al.‘s (2005) work shows that tannins form large, insoluble complexes when they interact with proteins. In each tube previously used for total polyphenols measurement (Folin–Ciocalteau method), 400 µL of egg albumin in acetate buffer was added. A gallic acid standard curve was used to convert absorbance into concentration. The tannin concentration (mg/mL) was estimated via the gallic acid standard curve: Tannin concentration (mg/mL) = [(AE − AB) − I] / S, where AE is the mean sample absorbance, AB the mean blank absorbance, I the intercept (0.089), and S the slope (0.561). Results are reported as mg gallic acid equivalents per g of dry extract.

### GC–MS (gas chromatography–mass spectroscopy) analysis

Chemical constituents were analyzed using a GC-MS system (Agilent 7000 GC coupled to an Agilent 7890 A MS). Separation was achieved on a HP-5MS capillary column. To facilitate the detection of polar phenolics and glycosides, samples were derivatized using MSTFA at 60 °C for 30 min. Identification was performed by comparing mass spectra with the NIST17/Wiley libraries. Retention indices (RI) were determined using a homologous series of n-alkanes (C8–C40) analyzed under identical conditions. Only compounds with a match factor > 85% were considered. Components with relative peak areas > [X]% were categorized as predominant (Keskes et al. 2017).

### Network pharmacology-based analysis

The most predominant compounds were subjected to pharmacologically based network analysis using the Stitch database http://stitch.embl.de/, and the Similarity Ensemble Approach (SEA) server database https://sea.bkslab.org/. Network visualizations were constructed using Cytoscape 3.10.2. Compounds are illustrated by nodes, and interaction is illustrated by edges.

### Nanoparticles preparation

*J. phoenicea* leaf extract-nanostructured lipid carriers (JP-NLCs) were prepared using a combined hot-melt dispersion and homogenization approach. Briefly, ethanol (90% v/v) was used as a solvent for *J. phoenicea* leaf extract, oleic acid, and Precirol^®^ATO5. The lipid phase consisted of an optimized blend of Precirol^®^ ATO 5 (solid lipid) and oleic acid (liquid lipid) in a 7:3 ratio, with the extract loaded at a concentration of 10 mg per gram of the total lipid matrix. The aqueous phase comprised 1% (w/v) Tween 80 in a final volume of 20 mL of deionized water. During the combined hot-melt dispersion and homogenization process, the lipid film was melted at 60 °C and combined with the aqueous phase under high-shear emulsification (10,000 rpm for 5 min) followed by ultrasonication (40% amplitude for 3 min) to achieve refined NLCs. In all experimental assays, blank NLCs (unloaded carriers) were prepared using the same lipid-to-surfactant ratios and processing parameters to serve as a negative control, ensuring observed effects were attributable to the extract and not the carrier components.

### Characterization of the synthesized JP-NLCs

JP-NLCs characteristics were assessed through the dynamic light scattering (DLS) technique (to determine size distribution, polydispersity index (PDI), and zeta potential). The encapsulated plant extract efficiency (EE%) was also assessed. Complementary ultrastructural assessment was conducted with transmission electron microscopy (TEM) (Shahin et al. 2022).

### Antioxidant activity of the prepared JP-NLCs

The free radical scavenging activity of the extract against the stable DPPH radical (0.004% in methanol) was determined according to the method of Braca et al. (2001). Briefly, 100 µL of DPPH solution was mixed with 100 µL of various sample concentrations (0.68–3.40 mg/mL) and incubated in the dark at room temperature for 30 min. The absorbance was measured at 517 nm using an Optima spectrophotometer, with three independent measurements. Scavenging activity was calculated as: % scavenging = [(AC − AE)/AC] × 100, where AC is the mean absorbance of the negative control, and AE is the mean absorbance of the extract. IC_50_ values were derived from the inhibition curve using Y = aX + c, where Y = 50, a is the slope, X is the sample concentration, and c is the intercept.

### Antimicrobial activity of the prepared JP-NLCs

Antimicrobial efficacy of the freshly prepared nanoparticles was assessed via disk diffusion, with MIC, MBC, and time-kill analyses conducted. For live/dead fluorescence assessments, a Leica DMI 6000 B FluoView confocal microscope (with TCS SP5) was employed. The system used a Kr/Ar laser at 488 nm, paired with a 514 nm long-pass emission filter to detect green signals from viable cells. A 580 nm beam splitter, along with a 520 nm long-pass (green) and a 590 nm long-pass (red) filter, enabled simultaneous dual-channel imaging, exploiting green fluorescence for live cells and red for dead cells (Elbhnsawi et al. 2023).

### Combination effect of JP-NLCs with the most commonly used antibiotics against the tested pathogens

Antibiotics tested included Cefotaxime, Amoxicillin, Cefoxitin, Ampicillin, Cefuroxime, Co-trimoxazole, Levofloxacin, Ciprofloxacin, Ceftriaxone, Meropenem, Tobramycin, Nitrofurantoin, Norfloxacin, Tigecycline, Cefoperazone/Sulbactam, Chloramphenicol, Ceftazidime, Piperacillin, Cefepime, Ampicillin/cloxacillin, Trimethoprim, Ofloxacin, Amikacin, Vancomycin, Amoxicillin/clavulanic acid, Tetracycline, and Rifampin. 20 µL of the nanoparticle (20 µg) was loaded onto each antibiotic disc and placed onto inoculated Mueller–Hinton agar. Interactions were classified as follows: additive if the combined effect equaled the sum of individual effects; antagonistic if the combined effect was less than the sum; synergistic if the combined effect exceeded it; and indifferent if there was no interaction (Foucquier and Guedj 2015).

Utilizing the broth microdilution checkerboard method, the synergistic potential of JP-NLCs combined with conventional antibiotics was evaluated based on the protocol described by Elnahas et al. (2021). The interactions were quantified using the Fractional Inhibitory Concentration Index (FICI), defining synergy as ≤ 0.5, additive as > 0.5–1, indifference as > 1–4, and antagonism as > 4.

Where FICI = FIC of agent A + FIC of agent B

$$\:\mathrm{F}\mathrm{I}\mathrm{C}\:=\:\frac{\mathrm{M}\mathrm{I}\mathrm{C}\:\mathrm{o}\mathrm{f}\:\mathrm{t}\mathrm{h}\mathrm{e}\:\mathrm{t}\mathrm{e}\mathrm{s}\mathrm{t}\mathrm{e}\mathrm{d}\:\mathrm{a}\mathrm{g}\mathrm{e}\mathrm{n}\mathrm{t}\:\mathrm{i}\mathrm{n}\:\mathrm{c}\mathrm{o}\mathrm{m}\mathrm{b}\mathrm{i}\mathrm{n}\mathrm{a}\mathrm{t}\mathrm{i}\mathrm{o}\mathrm{n}}{\mathrm{M}\mathrm{I}\mathrm{C}\:\mathrm{o}\mathrm{f}\:\mathrm{t}\mathrm{h}\mathrm{e}\:\mathrm{s}\mathrm{a}\mathrm{m}\mathrm{e}\:\mathrm{t}\mathrm{e}\mathrm{s}\mathrm{t}\mathrm{e}\mathrm{d}\:\mathrm{a}\mathrm{g}\mathrm{e}\mathrm{n}\mathrm{t}\:\mathrm{a}\mathrm{l}\mathrm{o}\mathrm{n}\mathrm{e}}.$$

### Anticancer effect of the newly synthesized JP-NLCs

Antitumor efficacy of the novel nanoparticles was evaluated using the A-549 lung carcinoma cell line. Cells were seeded at 5 × 10^4^ per well in 96-well plates and allowed to attach for 24 h. Four treatments were tested: crude *J. phoenicea* extract, JP-NLCs, 5-fluorouracil (5-FU), and a combination of equal volumes of the IC_50_ 5-FU and the IC_50_ of the prepared nanoparticles. Each agent was applied at ten serially diluted concentrations and incubated for 48 h. Cell viability was assessed by the MTT assay: 100 µL of phenol red–free RPMI 1640 was added, followed by 10 µL of 12 mM MTT stock (5 mg/mL in PBS) to every well. After 4 hrs at 37 °C and 5% CO_2_, 85 µL of medium was replaced with 50 µL DMSO (0.5%), mixed for 10 min at 37 °C, and absorbance was read at 590 nm (SunRise plate reader, TECAN). Viability was calculated as (ODt/ODc)×100, where ODt is the treated wells’ mean OD and ODc is the control mean OD. Dose–response curves were generated for each treatment, and IC_50_ values were estimated from the curves using GraphPad Prism (San Diego, CA, USA) (Mosmann 1983).

### Caspase enzymatic activity via fluorometric determination

Caspase enzymatic activity was quantified via fluorescence using specific fluorogenic substrates to delineate apoptotic pathways. Caspase-3 activity was assessed using Ac-DEVD-AFC, while initiator Caspase-9 and Caspase-8 activities were measured using Ac-LEHD-AFC and Ac-IETD-AFC, respectively (Alexis, Switzerland). Briefly, cellular proteins were extracted and allocated (95 µL per well) into Microlon ELISA plates. Substrates were added to a final concentration of 100 µM. Real-time fluorescence resulting from the cleavage and release of AFC was monitored at 37 °C using a microplate reader (Infinite M200, Tecan, Austria). All results and subsequent discussions were updated to reflect these specific substrate-protease pairings, ensuring that the biochemical interpretation of the intrinsic (Caspase-9) vs. extrinsic (Caspase-8) pathway activation is accurate (Xiao et al. 2012).

### Statistical analysis

The data obtained by the different afore-mentioned analysis methods were the mean ± SD of three trials which were statistically analyzed by an analysis of variance (ANOVA) via the GraphPad Prism 7.0d software (GraphPad Software Inc., San Diego, CA, USA). Statistical differences were designated as significant if p-values were less than 0.05 (*p ≤ 0.05), and highly significant if p-values were less than 0.01 (** *p* ≤ 0.01) or less than 0.001 (*** *p* ≤ 0.001).

## Results

### Antimicrobial activities of *Juniperus phoenicea* L. extracts

*J. phoenicea* L. leaves were extracted with ethanol and ethyl acetate one at a time using the maceration extraction technique. The resulting extract from each was tested against multidrug-resistant (MDR) microbes. As shown in Table 1, the inhibition zone (IZ) diameters against the tested microbes were marginally wider with ethanol extracts as compared to ethyl acetate extracts (range: 6.0–21.0 mm vs. 6.0–16.0 mm, respectively). Therefore, the ethanolic extract was preferred to conduct further analysis. The largest IZ diameter was recorded against *A. baumannii*. The MIC values of *J. phoenicea* L. leaves extracts were found to be in the ranges of 12.5–200 µg/mL and 25–200 µg/mL against all the tested MDR bacteria for ethanol and ethyl acetate, respectively.

**Table 1:** The antibacterial effect of the extracted leaf samples

Tested pathogens	Antibacterial effect			
	*J. phoenicea* L. leaves ethyl acetate extract		*J. phoenicea* L. leaves ethanolic extract	
	IZ (mm)	MIC (µg/mL)	IZ (mm)	MIC (µg/mL)
*E. coli*	16.0	50.0	18.5	25.0
MRSA	13.5	100.0	19.0	25.0
*P. mirabilis*	10.0	100.0	16.0	50.0
*A. baumannii*	15.5	25.0	21.0	12.5
*E. coli* 5922	6.0	200.0	18.0	25.0
MRSA 13300	11.0	100.0	16.0	50.0
*Enterobacter aerogenes*	6.0	200.0	6.0	200.0

### Measurements of the flavonoid, tannins, and total polyphenolic contents of the promising extract

Phytochemical analysis detected the different families of compounds existing in the plant by quantitative characterization reactions (Table S1). The total phenolic, flavonoid, and tannin contents in *J. phoenicea* L. were calculated from the regression equations of the calibration curves: y = 3.989x + 0.0211, R2 = 0.986; y = 0.211x + 0.1441, R2 = 0.892; and y = 0.115x + 0.1405, R^2^ = 0.98, respectively. The total tannins, polyphenolic, and flavonoid concentrations were 0.303 ± 0.005 µg/ml, 0.048 ± 0.005 mg/g, and 0.012 ± 0.007 mg/g, respectively.

#### GC–MS (gas chromatography–mass spectroscopy) analysis

**Fig. 1:**
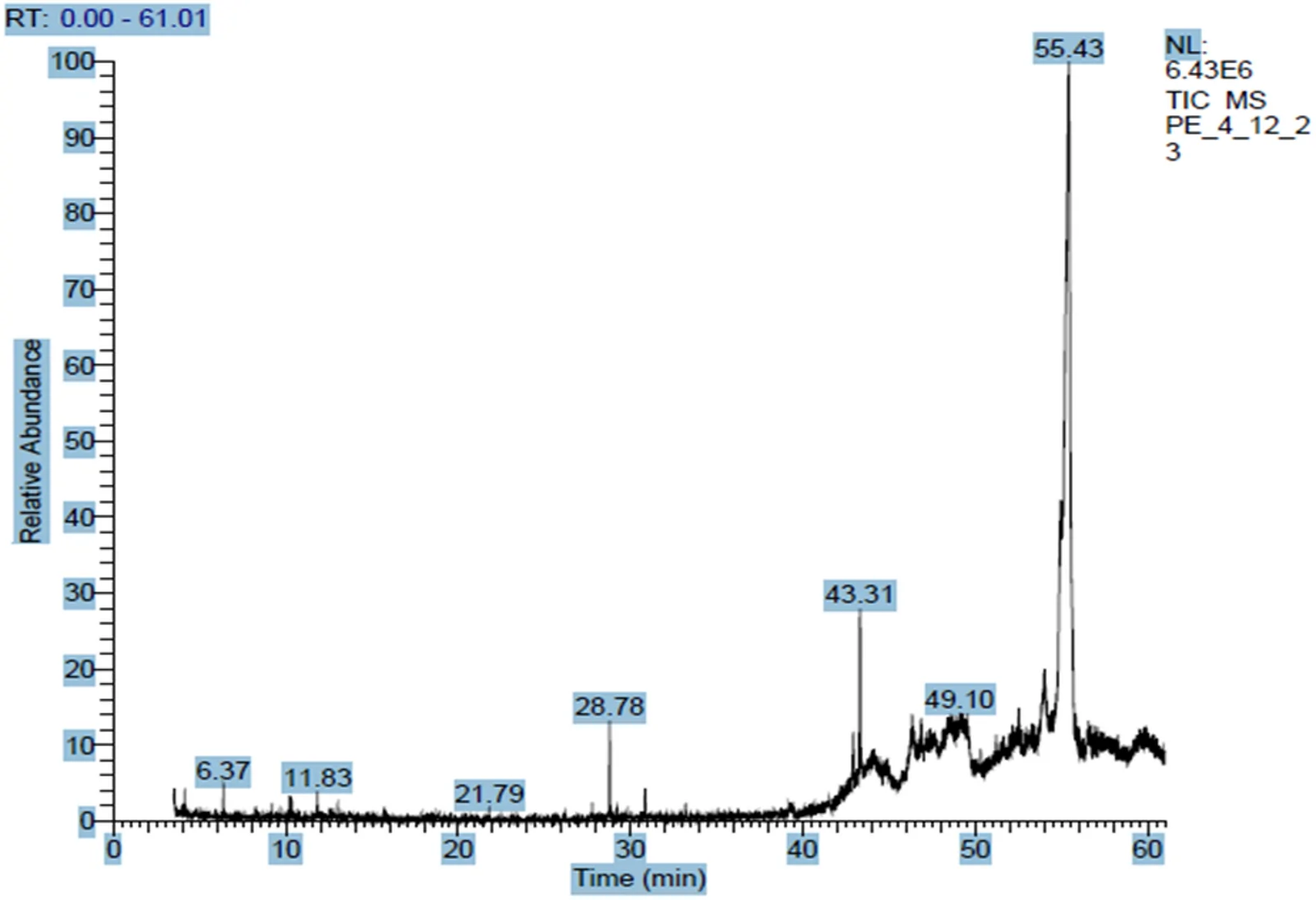
GC–MS chromatograms of *J. phoenicea* L. ethanolic extract

*J. phoenicea* L. ethanolic extract was prepared and analysed using GC–MS (Fig. 1). Apigenin 7-glucoside, α-pinene, and Cedrol were identified with 11.21, 23.49, and 10.93 area percentages, respectively (Table S2).

### Network pharmacology-based analysis

**Fig. 2:**
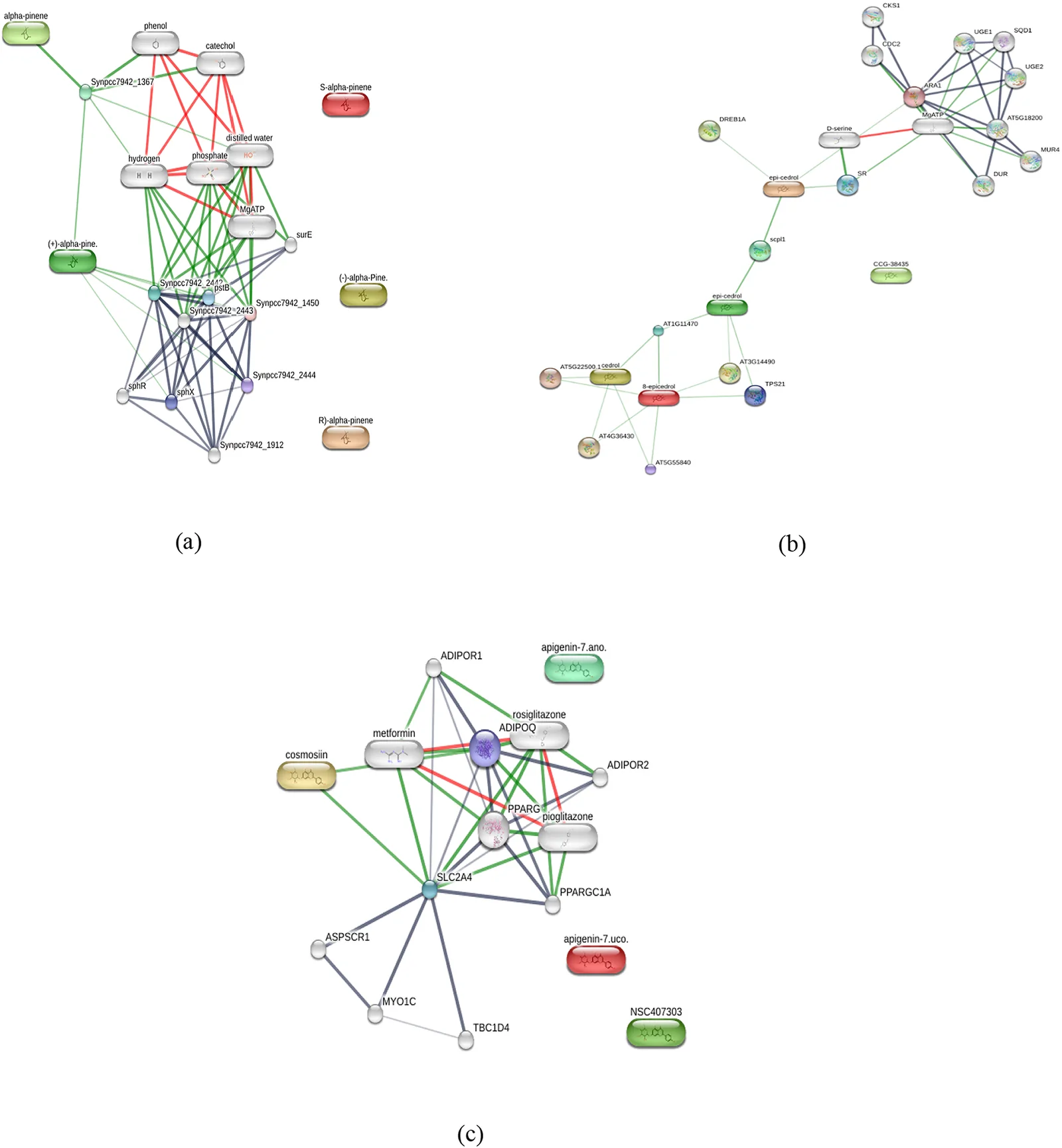
Network analysis of alpha-pinene (**a**), cedrol (b), and Apigenin 7-glucoside (**c**)

Targets were inferred by integrating human targets identified in the STITCH and SEA databases with reported juniper-derived compounds via a Cytoscape network (Fig. 2). The combined score quantified the interaction strength between each compound (Cedrol, α-pinene, and Apigenin 7-glucoside) and its corresponding targets. Filtering was applied so that only interactions with a minimum combined score of 0.9 were retained (Table S3). These computational findings are intended for hypothesis generation only. They provide a theoretical framework for how compounds like Cedrol, α-pinene, and Apigenin 7-glucoside might interact with cellular pathways but do not constitute proven pharmacological effects.

#### Characterization of the synthesized JP-NLCs

**Fig. 3:**
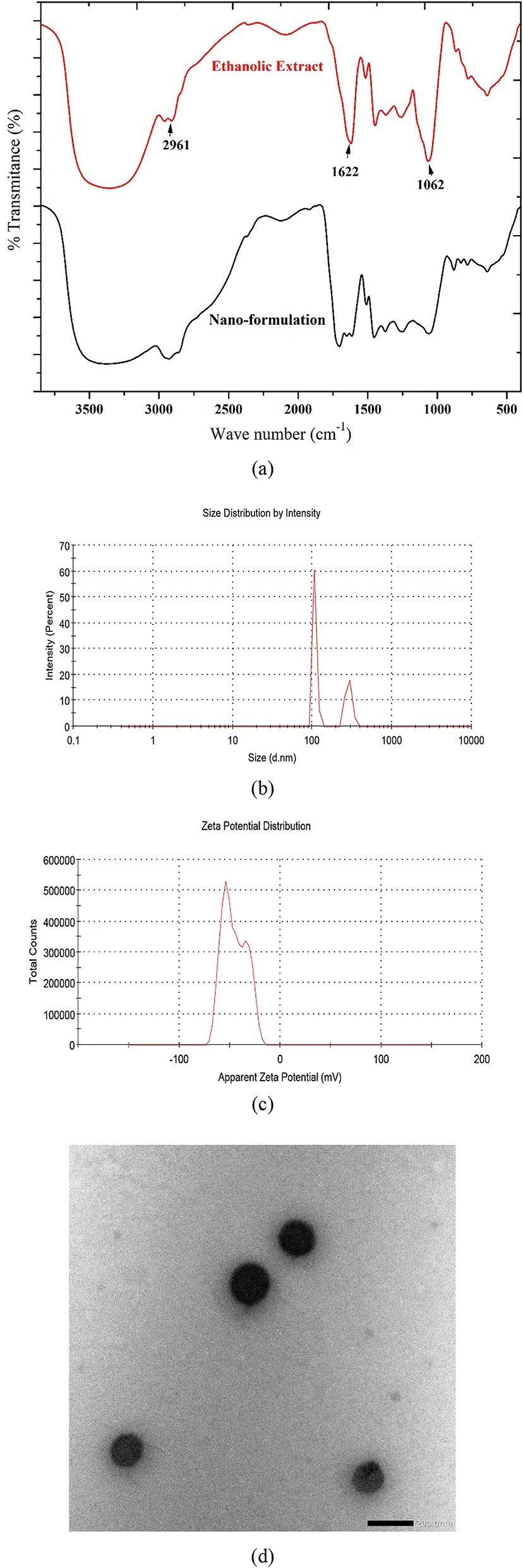
Nanoparticle characterizations where **a** IR spectroscopy of *J. phoenicea* L. ethanolic extract and JP-NLCs, **b** zeta size, **c** zeta potential and **d** TEM study of the synthesized JP-NLCs

*J. phoenicea* L. nanoparticles were newly synthesized through a combined hot-melt dispersion and homogenization approach. The recorded zeta vesicle size was 98.6 nm, while the PDI, zeta potential, EE%, and TEM recorded size were 0.23, + 48.5 mV, 92.4% and ~ 76 nm (range: ~60–100 nm), respectively. The nanoparticles were therefore considered homogeneous and stable. Moreover, Fourier Transform Infrared (FTIR) spectra analysis showed a broad, intense characteristic peak near 3430 cm^− 1^ due to O–H and N–H stretching vibration, together with a weak C–H stretching vibration band in the region of 2960 cm^− 1^. Furthermore, the band towards 1622 cm^− 1^ was attributed to the stretching vibration of C=O in *J. phoenicea* L. nanoparticles. Overall, the FTIR results show the successful nanoparticulate system as shown in Fig. 3. The nano-formulation shows a broader and more intense absorption band in the 3400 cm^−1^ region compared to the extract. This broadening suggests an increase in hydrogen bonding or the participation of more hydroxyl (–OH) groups from phenolic compounds and alcohols in stabilizing the nanoparticle surface. Moreover, the extract’s peak at 1622 cm^−1^ (C=O) shows altered intensity in the nano-formulation. Such shifts indicate that proteins or terpenoids acted as reducing agents, where carbonyl groups may have been oxidized to carboxylic acids during the reduction of metal ions. The retention of the 2960 cm^−1^ peak (C–H) with slight shifts or intensity variations confirms that the hydrophobic components of the extract remain associated with the nano-formulation as part of the protective capping layer (Pasieczna-Patkowska et al. 2025).

#### Antioxidant activity of the prepared nanoparticles

Total antioxidant DPPH free radical results showed that the prepared nanoformula showed increased antioxidant capacity with an IC_50_ value (mean ± SD) of 1491.41 ± 51.42 µg/mL as compared to the plant extract’s IC_50_ of 1542.77 ± 44.52 (Table S4).

#### Antimicrobial activity of the prepared nanoparticles

**Fig. 4:**
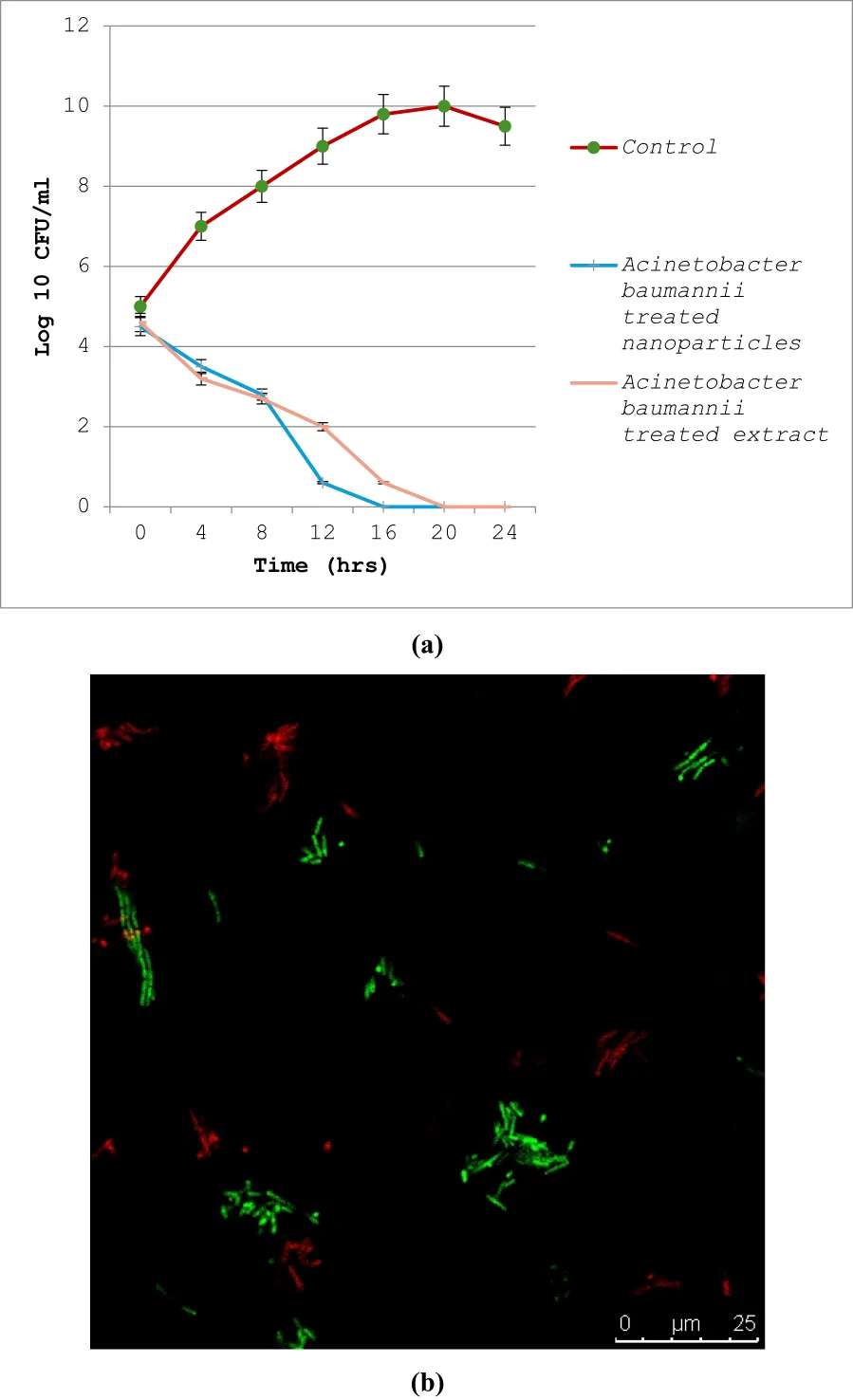
Time kill curve of treated cells (**a**) and CLSM study of microbially treated cells (merged image of dead red cells and viable green cells) (**b**)

As shown in Table 2, JP-NLCs resulted in an inhibition zone (IZ) with a 22 mm diameter against *A. baumannii*. MIC was 6.25 µg/mL, and the MBC value was 100 µg/mL. To test the inhibitory time for JP-NLC-treated bacterial cells, microbial lethality curves were evaluated. The findings illustrated in Fig. 4a indicate a consistent reduction in the growth of *A. baumannii* when treated with either nanoparticles or extract derived from *J. phoenicea L.*, till achieving complete eradication after 16 h and 20 h, respectively. Live/dead cell assay confirmed a reduction in viable microbial count after exposure to the nanoparticles. The increased dead cells (in red) as compared to the viable cells (in green) are demonstrated in Fig. 4b.

**Table 2:** Antimicrobial activity of JP-NLCs

Tested pathogens	IZ (mm)	MIC (µg/mL)	MBC (µg/mL)
*E. coli*	20.0	12.5	200.0
MRSA	18.0	12.5	200.0
*P. mirabilis*	19.0	25.0	100.0
*A. baumannii*	22.0	6.25	100.0
*E. coli* 5922	20.0	25.0	100.0
MRSA 13300	18.0	25.0	100.0
*Enterobacter aerogenes*	10.0	50.0	300.0

### Combination effect of JP-NLCs with the most commonly used effective antibiotics against the tested ***Acinetobacter baumannii***

The interactions of JP-NLCs with commonly known antibiotics were evaluated against *A. baumannii* using the disc diffusion method. As shown in Tables 3 and 4, a synergistic effect was demonstrated when combining the nanoparticles with Amoxicillin and Cefoxitin. On the other hand, the other combinations were antagonistic. This finding can be valuable in developing new strategies to overcome the ominous rise of antimicrobial resistance.

**Table 3:** Effects of combinations of JP-NLCs with commonly used antibiotics

Tested antibiotic	Inhibition zone diameter (mm)		
	Antibiotic alone	JP-NLCs	Combination
Cefotaxime	16.0	22.0	20.0
Amoxicillin	*6.0	22.0	30.0
Cefoxitin	*6.0	22.0	29.0
Ampicillin	10.0	22.0	18.0
Cefuroxime	*6.0	22.0	19.0
Co-trimoxazole	*6.0	22.0	18.0
Levofloxacin	*6.0	22.0	10.0
Ciprofloxacin	*6.0	22.0	10.0
Ceftriaxone	*6.0	22.0	10.0
Meropenem	*6.0	22.0	12.0
Tobramycin	18.0	22.0	35.0
Nitrofurantoin	8.0	22.0	13.0
Norfloxacin	*6.0	22.0	13.0
Tigecycline	18.0	22.0	20.0
Cefoperazone/Sulbactam	*6.0	22.0	10.0
Chloramphenicol	15.0	22.0	19.0
Ceftazidime	*6.0	22.0	10.0
Piperacillin	15.0	22.0	20.0
Cefepime	*6.0	22.0	10.0
Ampicillin/cloxacillin	15.0	22.0	25.0
Trimethoprim	*6.0	22.0	10.0
Ofloxacine	20.0	22.0	32.0
Amikacin	15.0	22.0	37.0
Vancomycin	15.0	22.0	20.0
Amoxicillin/clavulonic acid	10.0	22.0	16.0
Tetracycline	7.0	22.0	13.0
Rifampin	9.0	22.0	11.0

*6.0 mm zones equal to the disk diameter (no inhibition)

**Table 4:** Checkerboard analysis of the synthesized nanoparticles with the most potent antibiotics

Antibacterial agent	MIC (µg/mL)		FIC	FICI	Interpretation
	Single drug	Combined drug			
JP-NLCs	6.25	1.56	0.25	–	–
Amoxicillin	32.0	4.0	0.125	0.375	Synergy
Cefoxitin	16.0	4.0	0.25	0.50	Synergy
Tobramycin	8.0	4.0	0.50	0.75	Additive
Amikacin	4.0	2.0	0.50	0.75	Additive

Interpretation Criteria: FICI 0.5 indicates synergy; 0.5 < FICI 1.0 indicates additivity; 1.0 < FICI 4.0 indicates indifference; FICI > 4.0 indicates antagonism. All mean values were statistically significant (*p* < 0.05) compared to individual agent MICs

### Anticancer effect of the newly synthesized JP-NLCs

**Fig. 5:**
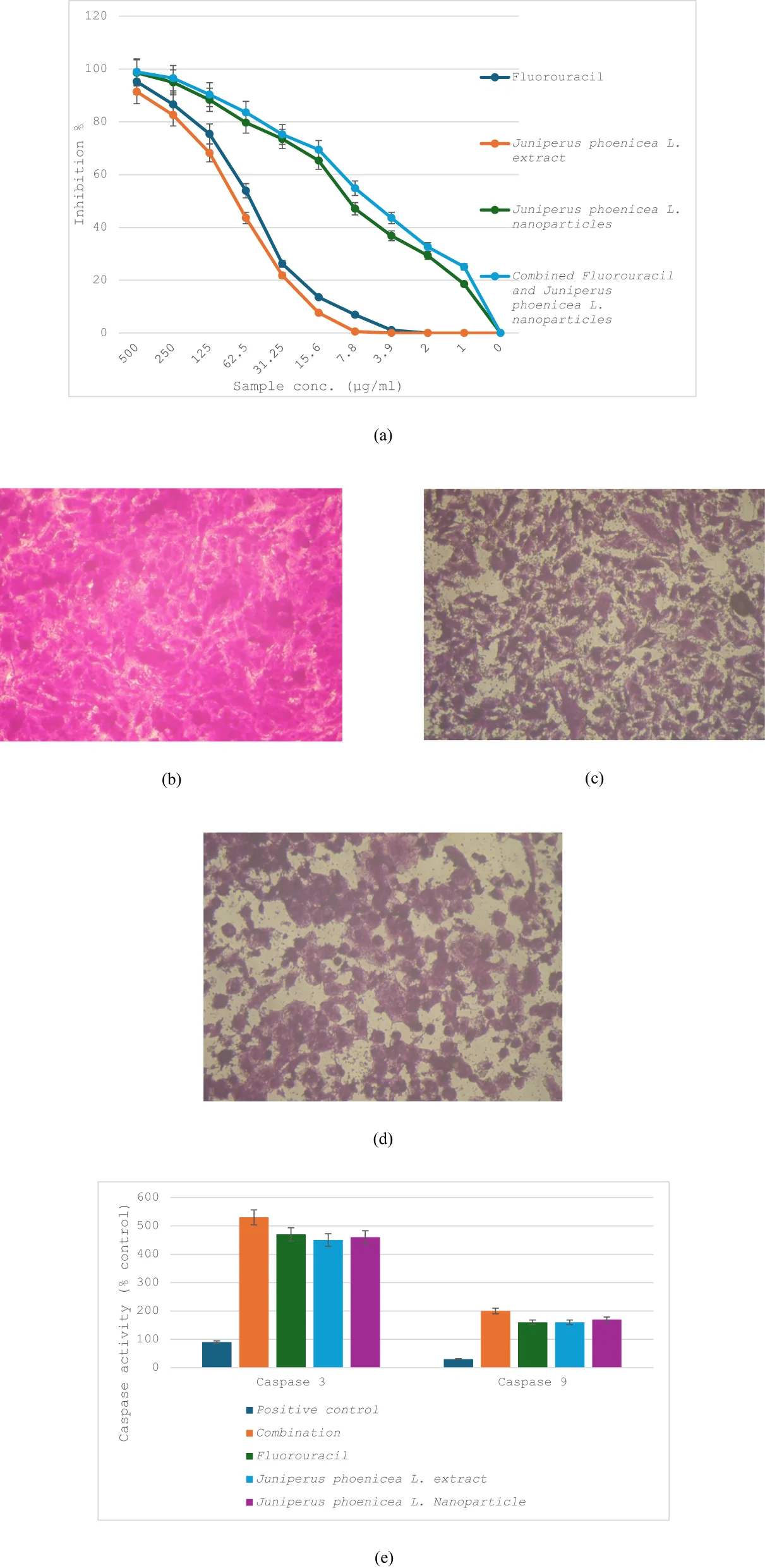
Inhibitory percentage against A-549 cells (**a**), Control untreated cells (**b**), Fluorouracil treated cells (**c**), Combined Fluorouracil/JP-NLCs (**d**), and caspase activity percentage (**e**)

Inhibitory activity using *J. phoenicea* L. extract against Lung carcinoma cells (A-549) was detected using MTT assay for 48 h, with IC_50_ reaching 78.77 ± 2.03 µg/ml. Evaluation of cytotoxicity against the lung cancer cell line presented the prepared nanoparticles’ magnificent inhibitory effect in comparison to the plant extract. To our surprise, the synthesized JP-NLCs showed superior activity over the standard treatment (Fluorouracil), which had an IC_50_ value equaled 58.11 ± 1.29 µg/ml. Upon testing the JP-NLCs, the detected IC_50_ was 9.05 ± 0.49 µg/ml (an 8.7-fold increase in the inhibitory effect compared to the crude extract). Moreover, in a combined study between Fluorouracil and JP-NLCs, significant synergy was detected with an IC_50_ reached 6.12 ± 0.41 µg/ml (Table S5, Fig. 5).

To evaluate the therapeutic synergy of the formulations, we utilized CalcuSyn software to determine the Combination Index (CI) and Dose Reduction Index (DRI). The DRI quantifies the fold-reduction in dosage required for an individual drug to maintain its efficacy when integrated into a combination. The combination of Drug Fluorouracil and Drug JP-NLCs was evaluated using the Chou-Talalay method to determine the nature of their pharmacological interaction. The CI analysis revealed a biphasic dose-response relationship. At low fractional inhibition (Fraction Affected (ƒ_a_) < 0.25), the combination exhibited moderate antagonism (CI > 1.0). However, a clear synergistic trend emerged at higher effect levels (ƒ_a_ = 0.42 to 0.76), achieving a peak synergistic effect at ƒ_a_ = 0.76 with a CI value of 0.59. This synergy is further supported by the Dose Reduction Index (DRI), which indicates that at the 76% effect level, the doses of Drug JP-NLCs and Drug Fluorouracil can be reduced by 10.34-fold and 2.02-fold, respectively, compared to their individual applications. These results (Table S6) suggest that the Fluorouracil + JP-NLCs combination is highly effective at therapeutic concentrations, offering the potential for enhanced efficacy with reduced dose-related toxicity. At the extreme effect level (ƒ_a_ = 0.96), the interaction reverted to antagonism (CI = 1.14), suggesting a saturation point for the synergistic mechanism.

## Discussion

Natural products offer a promising avenue to combat MDR microbes by targeting diverse bacterial pathways and reducing the likelihood of resistance development. In the present investigation, the ethanolic extract of *J. phoenicea* L. leaves showed higher IZ diameter when tested against *A. baumannii* when compared to the ethyl acetate extract. The extract was considered active when it induced an inhibition zone of more than or equal to 10 mm but was considered non-active when it induced inhibition zones of less than 7 mm (Tekwu et al. 2012). As explained by Khan et al. (2011), the antimicrobial activity demonstrated can be attributed to the active secondary metabolites found in the extracts. The present findings are consistent with Derwich et al. (2010), who reported that *E. coli* was the most susceptible strain tested to the leaves’ oil of Moroccan *J. phoenicea*, while *S. aureus* was shown to be resistant to the same oil. Another study investigated the therapeutic potential of *J. phoenicea* leaves by comparing the efficiency of three solvent systems: 70% methanol, ethanol, and acetone. Beyond quantifying the total phenolic and flavonoid yields, they evaluated the extracts’ antioxidant capacity via DPPH radical scavenging and ferric reducing power. While all extracts demonstrated robust antibacterial properties against five distinct Gram-positive and Gram-negative strains—particularly at concentrations between 20% and 40%—70% acetone emerged as the superior solvent. It consistently yielded the highest concentration of bioactive compounds and exhibited the most potent antioxidant and reduced performance (Elmhdwi et al. 2015).

The total tannins, polyphenolic, and flavonoid concentrations of *J. phoenicea* L. leaves were 0.303 ± 0.005 µg/ml, 0.048 ± 0.005 mg/g, and 0.012 ± 0.007 mg/g, respectively. The capacity of these secondary metabolites to suppress microbial growth has made them valuable as potent natural compounds and phytochemicals (Amira et al. 2012). When combined with antibiotics, these substances may have a synergistic impact that increases their antibacterial efficacy, in addition to acting as biologically active substances on their own against microorganisms (Barakat et al. 2020). GC–MS analysis of *J. phoenicea* L. ethanolic extract revealed the presence of Apigenin 7-glucoside, α-pinene, and Cedrol. According to Al-Khlifeh et al. (2021), the compositions of the extracts of *J. phoenicea* exhibit quantitative variations based on geographical origin and distribution among various tree sections. Across the *Juniperus* species, the volatile oils of leaves and berries tell a distinct chemical story. For most, the narrative is driven by monoterpene hydrocarbons, which claim a dominant share of the profile—ranging from a notable 39.7% in *J. oxycedrus* to a staggering 79.0% in *J. macrocarpa*. At the heart of this profile is α-pinene, the prevailing compound across the genus, averaging 41.3% in *J. macrocarpa*, 43.0% in *J. oxycedrus*, and 27.6% in *J. communis*. However, *J. deltoides* breaks the mold, pivoting away from this trend with limonene emerging as its primary chemical constituent at 20.0% (Najar et al. 2020).

It is well acknowledged among researchers that the leaf extract of *J. phoenicea* mostly consists of α-pinene and other pinane derivatives. α-pinene is the main component, followed by cedrol as the second most significant component. Indeed, the antibacterial effects of medicinal plants are challenging to elucidate. That is because many elements are recognized to contribute to and interact mutually within it. The antibacterial impact of the *J. phoenicea* extract may be attributed, at least in part, to the primary components of its extract, including α-pinene and cedrol. These constituents have been shown to have antibacterial properties. This implies that the α-pinene and cedrol may have a significant role in augmenting the bioactivity of *J. phoenicea* (Althunibat et al. 2016).

Network pharmacology-based analysis of Cedrol, α-pinene, and Apigenin 7-glucoside and its corresponding targets was assessed. For example, Apigenin 7-glucoside (A7G, also known as apigetrin) is a plant-derived flavonoid glycoside with notable antibacterial and anticancer properties. Recent research has focused on its molecular mechanisms, revealing both direct effects on cancer cell survival and bacterial growth, as well as modulation of key cellular pathways. Apigenin 7-glucoside exerts its antibacterial effects by disrupting biofilms, increasing membrane permeability, and inhibiting key bacterial enzymes, while its anticancer activity involves cell cycle arrest, apoptosis induction, and inhibition of survival pathways (Gok et al. 2025; Kamel et al. 2025; Li et al. 2024; Zhong et al. 2023). These multifaceted mechanisms highlight A7G’s promise as a natural therapeutic agent for both infectious diseases and cancer. The network pharmacology analysis was conducted based on the putative identification of compounds via GC-MS. While RI and mass spectral matching provide high confidence, the exclusion of non-derivatizable high-molecular-weight compounds by GC-MS means the network represents the volatile and semi-volatile fraction of the extract. Further LC-MS/MS studies are warranted to capture the full polar metabolome.

Notably, to date, there is no published report on *J. phoenicea* extract–loaded nanocarriers (NLCs, solid lipid nanoparticles, or chitosan-based systems). However, De Barros et al. (2022) reported that the intrinsic bioactivity of NLCs derived from natural plant oils can be amplified through the encapsulation of bioactive cargos, and these enhanced carriers, in turn, can augment the topical administration of therapeutics. Quercetin (QR), a quintessential plant flavonoid, exhibits broad-spectrum antibacterial effects. The primary aim of De Barros et al. (2022) work was to probe the synergistic potential of loading NPO-NLCs with quercetin (QR-NPO-NLCs) as a topical vehicular system for treating cutaneous bacterial infections. They engineered five NLC formulations by varying the lipid/oil constituents—sunflower, olive, corn, coconut, and castor oils. The optimized NLCs demonstrated sub-200 nm hydrodynamic diameters with a zeta potential around 40 mV, indicating robust colloidal stability.

Total antioxidant DPPH free radical results showed that the prepared nanoformula showed increased antioxidant capacity with an IC_50_ 1491.41 ± 51.42 µg/mL. These findings are in line with the work by Al-Mustafa et al. (2021), who demonstrated the presence of phenols, alkaloids, flavonoids, terpenoids, anthraquinones, and glycosides in the natural extracts of *J. Phoenicea* (JP) and *Calicotome Villosa* (CV) leaves. The levels of flavonoids and flavonols were substantially greater in JP as compared to CV (*p* < 0.05), while the amount of phenolic content was similar in both. In addition, the scavenging capacity of JP extract for DPPH radicals was found to be superior to that of CV extract, with IC_50_ values of 11.1 µg/ml and 15.6 µg/ml, respectively. However, both extracts showed comparable antioxidant capabilities by the FRAP experiment, which were concentration dependent (Al-Mustafa et al. 2021).

JP-NLCs resulted in a consistent reduction in the growth of *A. baumannii* when treated with nanoparticles, achieving complete eradication after 16 h. Live/dead cell assay confirmed a reduction in viable microbial count after exposure to the nanoparticles. This antimicrobial action of nanoparticles from *J. phoenicea L.* was previously discussed by Ramdani et al. (2013), who mentioned that the efficacy against both Gram-positive and Gram-negative bacteria is achieved by compromising their cell membranes.

The formulated *J. phoenicea* NLCs (98.6 nm; PDI 0.23; +48.5 mV; EE 92.4%; TEM 76.2 nm) fall in the small, monodisperse, strongly cationic range considered optimal for colloidal stability and biological interaction. PDI < 0.3 generally reflects a homogeneous population of lipid nanocarriers (De Barros et al. 2021). Zeta potentials ≥ |30| mV usually prevent aggregation; NLC systems with − 36 to − 66 mV remain stable and show good antimicrobial performance (De Barros et al. 2021) (Table 5).

**Table 5:** Comparison with other herbal NLCs

System	Size (nm)	Zeta (mV)	Antimicrobial outcome	References
Propolis NLCs	~ 42–44	Negative (not specified)	~ 2× higher antibacterial effect vs. crude extract, broad-spectrum	Elkhateeb et al. ( 2022)
NLC–natural plant oils	155–240	−36 to − 66	Complete growth inhibition at non-cytotoxic doses (strain-dependent)	De Barros et al. (2021)
JP-NLC	98.6	+ 48.5	Complete eradication of the microbial growth after 16 h	Current work

The formulated nanoparticles are somewhat larger than propolis NLCs but smaller than many NLC–oil systems, with much higher surface charge (+ 48.5 mV), which should further enhance electrostatic interaction with the negatively charged outer membrane of *A. baumannii*. Cationic vesicles and polymers show enhanced binding to Gram-negative bacteria, causing clustering, morphological distortion, and membrane disruption via electrostatic attraction to anionic membranes (Banerjee et al. 2024).

For *A. baumannii*, agents that permeabilize the membrane (antimicrobial peptides, repurposed drugs) induce depolarization, ATP loss, and ROS accumulation, leading to rapid killing and biofilm inhibition (Luo et al. 2025). By damaging the outer membrane and disturbing proton motive force, such agents can compromise efflux pumps and barrier function, which is a plausible route to sensitizing carbapenem- or ceftazidime-resistant *A. baumannii* (Yuan et al. 2025), though this has not been tested with *J. phoenicea* NLCs specifically. Given their small size, low PDI, and strong positive zeta potential, *J. phoenicea*–loaded NLCs are well-positioned to bind and disrupt *A. baumannii* membranes and may help overcome resistance, but direct studies on this specific formulation and species are still needed.

The interactions of JP-NLCs with commonly known antibiotics showed a synergistic effect with Amoxicillin and Cefoxitin. This finding can be valuable in developing new strategies to overcome the ominous rise of antimicrobial resistance. Synergism with a natural substance could allow the use of lower doses of antibiotics, while potentially reducing the development of resistance. Examples of that can be appreciated in the work done by Abreu et al. (2012) and Betts et al. (2014), who could isolate different biologically active phytochemicals from herbs. Those substances were found to exert their antimicrobial activity via different mechanisms. For instance, isopimarane, carnosic acid, reserpine, and EGCG were found to block bacterial efflux pumps, while corilagin and Tellimagrandin targeted the bacterial penicillin-binding proteins (PBP2a). Other substances, such as corilagin, gallic acid, and thymol, increased the outer membrane permeability, whereas Berberine and 5′-methoxyhydnocarpin were found to act on MDR microbes’ efflux pump.

The anticancer study demonstrates that Juniperus-derived formulations (plant extract and JP-NLCs) modulate apoptotic pathways in the human lung cancer cell line, as evidenced by significant reductions in Caspase 3/9 activity. The findings are particularly relevant to non-small cell lung cancer models, where targeting MCM and apoptotic regulators has therapeutic potential. The comparative efficacy against standard chemotherapeutics (e.g., fluorouracil) and the observed synergistic interactions (with amoxicillin/cefoxitin in antimicrobial assays, and with fluorouracil in anticancer assays) warrant further mechanistic exploration and in vivo validation to assess translational potential. For instance, cedrol, a single bioactive constituent isolated from *J. chinensis*, markedly downregulates minichromosome maintenance (MCM) protein expression in human lung carcinoma A549 cells, reduces A549 cell viability, induces G1-phase cell cycle arrest, and amplifies apoptosis (Yun et al. 2022).

Chien et al. (2025) reported that the combination of *J. indica* Bertol extract (JIB) with 5-fluorouracil (5-FU) exhibits a notable enhancement in anti-tumor activity against oral squamous cell carcinoma (OSCC), surpassing the effects observed with either agent alone in OECM-1 cells (*p* < 0.05). Co-treatment with JIB and 5-FU markedly arrested the cell cycle, correlated with reduced expression of CKD2/cyclin A and CDK4/cyclin D1 (*p* < 0.05). Additionally, the dual therapy promoted apoptosis via activation of caspases and modulation of the Bax/caspase-9/caspase-3 axis. Importantly, JIB impeded the regrowth of cells previously exposed to 5-FU and reduced the emergence of 5-FU–resistant populations during OSCC treatment (*p* < 0.05).

To be concluded, this study delineates the biological profile of *J. phoenicea* L. and presents a novel nanoparticle formulation derived from this plant. The present findings support further exploration of *J. phoenicea* L. nanoparticles as multifunctional agents with antimicrobial, antioxidant, and anticancer synergy.

### Impact statement

The study demonstrates that *J. phoenicea* L. possesses potent antibacterial, antioxidant, and anticancer properties, and that its ethanolic extract can be leveraged to synthesize novel nanoparticles with superior bioactivity. The findings open avenues for developing plant-based nanomedicines that can augment existing antibiotics and chemotherapeutics, potentially address antimicrobial resistance, and improve cancer treatment efficacy. The dual antimicrobial/anticancer profile of the nanoparticles offers a platform for multi-target therapeutic approaches. However, the current results are based on in vitro assays; in vivo efficacy, detailed mechanistic insights, selectivity and toxicity profiles remain to be established before clinical translation.

## Supplementary Information

Below is the link to the electronic supplementary material.

Supplementary Material 1

## Data Availability

All the original data are available upon reasonable request for correspondence authors.
