# Supplementary material for: Juniperus phoenicea extract-loaded nanostructured lipid carriers for potent antimicrobial and anticancer combination therapy
Source: AMB Express. 2026 Sep 4;16:88. doi: 10.1186/s13568-026-02054-0 (PMC13550337; doi:10.1186/s13568-026-02054-0)
Supplement: Supplementary file 1 [file 13568_2026_2054_MOESM1_ESM.docx]

***Juniperus phoenicea* extract-loaded nanostructured lipid carriers (NLCs) for Potent Antimicrobial and Anticancer Combination Therapy**

Passant A. Ismail^1*^, Bassma H. Elwakil^2^, Mohamed Hagar^3^, Zakia A. Olama^1^

^1^ Botany & Microbiology Department, Faculty of Science, Alexandria University, Alexandria 21500, Egypt

^2^ Medical Laboratory Technology Program, Faculty of Applied Health Sciences Technology, Galala University, Suez, Egypt

^3^ Chemistry Department, Faculty of Science, Alexandria University, Alexandria 21500, Egypt

*Correspondence: [**passant.abdelmksoud_pg@alexu.edu.eg**](mailto:passant.abdelmksoud_pg@alexu.edu.eg)

**Table S1.** Total polyphenolic, flavonoid, and tannin concentrations of the extract

| Tested Extract | Total tannins (ug/ml) | Total polyphenolic (mg/g) | Total flavonoid (mg/g) |
| --- | --- | --- | --- |
| Juniperus phoenicea L. | 0.303 ± 0.00502 | 0.0478 ± 0.0049 | 0.0122 ± 0.00724 |

**Table S2.** The major expected compounds of *Juniperus phoenicea* L. ethanolic extract

| **RT** | **Area %** | **Compound Name** |
| --- | --- | --- |
| 6.37 | 0.76 | 3-Cyclohexen-1-ol, 4-methyl-1-(1-methylethyl)-, acetate |
| 10.33 | 0.50 | L-Citrulline |
| 11.83 | 0.89 | Vitamin A aldehyde |
| 28.78 | 11.21 | Apigenin 7-glucoside |
| 30.84 | 0.74 | Cedrol |
| 43.31 | 23.49 | α-pinene |
| 43.81 | 0.76 | Cyclopentadecanol |
| 44.00 | 0.65 | Hexadecanoic acid, 2,3-dihydroxypropyl ester |
| 44.21 | 0.64 | Ergotaman-3',6',18-trione, 12'-hydroxy-2'-methyl-5'-(phenylmethyl)-, (5'à)- |
| 46.33 | 2.39 | Hydrocortisone Acetate |
| 46.68 | 0.50 | 5-Amino-2-methoxyphenol |
| 46.84 | 1.30 | Ricinoleic acid |
| 49.27 | 10.93 | Cedrol |
| 49.38 | 0.55 | Chlorpromazine |
| 49.60 | 0.92 | Carmine acid |

**Table S3.** Library RI of the most predominant compounds

| **Peak** | **RT (min)** | **Calculated RI** | **Library RI** | **Compound** | **Match Score** | **Rel. Area (%)** |
| --- | --- | --- | --- | --- | --- | --- |
| 1 | 5.24 | 936 | 939 |   -Pinene | 910 | 18.4 |
| 2 | 28.15 | 2145 | 2152 | Apigenin | 865 | 12.2 |

**Table S3.** Network analysis

| Cedrol | | | | | | |
| --- | --- | --- | --- | --- | --- | --- |
| node1 | node2 | node1 accession | node2 accession | node1 annotation | node2 annotation | score |
| UGE2 | AT5G18200 | AT4G23920.1 | AT5G18200.1 | UDP-D-glucose/UDP-D-galactose 4-epimerase 2; Catalyzes the interconversion between UDP-glucose and UDP-galactose. Cooperates with UGE3 in pollen development and with UGE4 in cell wall carbohydrate biosynthesis and growth | galactose-1-phosphate uridylyltransferase | 0.999 |
| UGE1 | AT5G18200 | AT1G12780.1 | AT5G18200.1 | UDP-D-glucose/UDP-D-galactose 4-epimerase 1; Catalyzes the interconversion between UDP-glucose and UDP-galactose and the interconversion between UDP-arabinose and UDP-xylose. Plays a role in D-galactose detoxification | galactose-1-phosphate uridylyltransferase | 0.999 |
| SQD1 | AT5G18200 | AT4G33030.1 | AT5G18200.1 | UDP-sulfoquinovose synthase; Involved in the biosynthesis of sulfolipids found in thylakoid membranes. Converts UDP-glucose and sulfite to the sulfolipid head group precursor UDP-sulfoquinovose | galactose-1-phosphate uridylyltransferase | 0.999 |
| MUR4 | AT5G18200 | AT1G30620.1 | AT5G18200.1 | MURUS 4; Acts as a UDP-D-xylose 4-epimerase but lacks both UDP-D- glucose and UDP-D-glucuronic acid 4-epimerase activities in vitro | galactose-1-phosphate uridylyltransferase | 0.999 |
| DUR | AT5G18200 | AT5G44480.1 | AT5G18200.1 | DEFECTIVE UGE IN ROOT | galactose-1-phosphate uridylyltransferase | 0.999 |
| CKS1 | ARA1 | AT2G27960.1 | AT4G16130.1 | cyclin-dependent kinase-subunit 1; Associates with cyclin-dependent kinases (CDKs) and plays an essential role in the regulation of the cell cycle that affects plant growth rate. May inhibit both the G1/S and G2/M phases | arabinose kinase; Arabinose kinase. Involved in the salvage pathway which converts free L-arabinose to UDP-L-arabinose. May play a role in arabinose transport | 0.999 |
| CKS1 | CDC2 | AT2G27960.1 | AT3G48750.1 | cyclin-dependent kinase-subunit 1; Associates with cyclin-dependent kinases (CDKs) and plays an essential role in the regulation of the cell cycle that affects plant growth rate. May inhibit both the G1/S and G2/M phases | cell division control 2; Involved in the control of the cell cycle. Essential for both G1/S and G2/M (mitosis) phase transitions. Functions in cell morphogenesis as well as cell proliferation. Required for cell division (entry into mitosis) of the generative cell in male gametogenesis | 0.999 |
| CDC2 | CKS1 | AT3G48750.1 | AT2G27960.1 | cell division control 2; Involved in the control of the cell cycle. Essential for both G1/S and G2/M (mitosis) phase transitions. Functions in cell morphogenesis as well as cell proliferation. Required for cell division (entry into mitosis) of the generative cell in male gametogenesis | cyclin-dependent kinase-subunit 1; Associates with cyclin-dependent kinases (CDKs) and plays an essential role in the regulation of the cell cycle that affects plant growth rate. May inhibit both the G1/S and G2/M phases | 0.999 |
| CDC2 | ARA1 | AT3G48750.1 | AT4G16130.1 | cell division control 2; Involved in the control of the cell cycle. Essential for both G1/S and G2/M (mitosis) phase transitions. Functions in cell morphogenesis as well as cell proliferation. Required for cell division (entry into mitosis) of the generative cell in male gametogenesis | arabinose kinase; Arabinose kinase. Involved in the salvage pathway which converts free L-arabinose to UDP-L-arabinose. May play a role in arabinose transport | 0.999 |
| AT5G18200 | SQD1 | AT5G18200.1 | AT4G33030.1 | galactose-1-phosphate uridylyltransferase | UDP-sulfoquinovose synthase; Involved in the biosynthesis of sulfolipids found in thylakoid membranes. Converts UDP-glucose and sulfite to the sulfolipid head group precursor UDP-sulfoquinovose | 0.999 |
| AT5G18200 | UGE2 | AT5G18200.1 | AT4G23920.1 | galactose-1-phosphate uridylyltransferase | UDP-D-glucose/UDP-D-galactose 4-epimerase 2; Catalyzes the interconversion between UDP-glucose and UDP-galactose. Cooperates with UGE3 in pollen development and with UGE4 in cell wall carbohydrate biosynthesis and growth | 0.999 |
| AT5G18200 | MUR4 | AT5G18200.1 | AT1G30620.1 | galactose-1-phosphate uridylyltransferase | MURUS 4; Acts as a UDP-D-xylose 4-epimerase but lacks both UDP-D- glucose and UDP-D-glucuronic acid 4-epimerase activities in vitro | 0.999 |
| AT5G18200 | DUR | AT5G18200.1 | AT5G44480.1 | galactose-1-phosphate uridylyltransferase | DEFECTIVE UGE IN ROOT | 0.999 |
| AT5G18200 | UGE1 | AT5G18200.1 | AT1G12780.1 | galactose-1-phosphate uridylyltransferase | UDP-D-glucose/UDP-D-galactose 4-epimerase 1; Catalyzes the interconversion between UDP-glucose and UDP-galactose and the interconversion between UDP-arabinose and UDP-xylose. Plays a role in D-galactose detoxification | 0.999 |
| ARA1 | CKS1 | AT4G16130.1 | AT2G27960.1 | arabinose kinase; Arabinose kinase. Involved in the salvage pathway which converts free L-arabinose to UDP-L-arabinose. May play a role in arabinose transport | cyclin-dependent kinase-subunit 1; Associates with cyclin-dependent kinases (CDKs) and plays an essential role in the regulation of the cell cycle that affects plant growth rate. May inhibit both the G1/S and G2/M phases | 0.999 |
| ARA1 | CDC2 | AT4G16130.1 | AT3G48750.1 | arabinose kinase; Arabinose kinase. Involved in the salvage pathway which converts free L-arabinose to UDP-L-arabinose. May play a role in arabinose transport | cell division control 2; Involved in the control of the cell cycle. Essential for both G1/S and G2/M (mitosis) phase transitions. Functions in cell morphogenesis as well as cell proliferation. Required for cell division (entry into mitosis) of the generative cell in male gametogenesis | 0.999 |
| AT5G18200 | ARA1 | AT5G18200.1 | AT4G16130.1 | galactose-1-phosphate uridylyltransferase | arabinose kinase; Arabinose kinase. Involved in the salvage pathway which converts free L-arabinose to UDP-L-arabinose. May play a role in arabinose transport | 0.998 |
| ARA1 | AT5G18200 | AT4G16130.1 | AT5G18200.1 | arabinose kinase; Arabinose kinase. Involved in the salvage pathway which converts free L-arabinose to UDP-L-arabinose. May play a role in arabinose transport | galactose-1-phosphate uridylyltransferase | 0.998 |
| SR | D-serine | AT4G11640.1 | 5951 | serine racemase; Catalyzes the synthesis of D-serine from L-serine. Has dehydratase activity towards both L-serine and D-serine. Displays high substrate specificity for L-serine, whereas L-alanine, L- arginine, and L-glutamine were poor substrates | D-serine | 0.995 |
| D-serine | SR | 5951 | AT4G11640.1 | D-serine | serine racemase; Catalyzes the synthesis of D-serine from L-serine. Has dehydratase activity towards both L-serine and D-serine. Displays high substrate specificity for L-serine, whereas L-alanine, L- arginine, and L-glutamine were poor substrates | 0.995 |
| Alpha-pinene | | | | | | |
| node1 | node2 | node1 accession | node2 accession | node1 annotation | node2 annotation | score |
| pstB | Synpcc7942_2442 | Synpcc7942_2441 | Synpcc7942_2442 | phosphate transporter ATP-binding protein; Part of the ABC transporter complex PstSACB involved in phosphate import. Responsible for energy coupling to the transport system (By similarity) | phosphate ABC transporter permease | 0.999 |
| pstB | Synpcc7942_2443 | Synpcc7942_2441 | Synpcc7942_2443 | phosphate transporter ATP-binding protein; Part of the ABC transporter complex PstSACB involved in phosphate import. Responsible for energy coupling to the transport system (By similarity) | phosphate ABC transporter permease | 0.999 |
| phosphate | MgATP | 1003 | 5957 | Phosphoric acid (also known as orthophosphoric acid or phosphoric(V) acid) is a mineral (inorganic) acid having the chemical formula H3PO4. Orthophosphoric acid molecules can combine with themselves to form a variety of compounds which are also referred to as phosphoric acids, but in a more general way. The term "phosphoric acid" can also refer to a chemical or reagent consisting of phosphoric acids, such as "pyrophosphoric acid" or "triphosphoric acid", but usually orthophosphoric acid.; Orthophosphoric acid refers to "phosphoric acid", which is the IUPAC name for this compound. The prefix "ortho" is used to distinguish the acid from related phosphoric acids, called polyphosphoric acids. Orthophosphoric acid is a non-toxic acid, which, when pure, is a solid at room temperature and pressure. | MgATP | 0.999 |
| Synpcc7942_2444 | Synpcc7942_2442 | Synpcc7942_2444 | Synpcc7942_2442 | phosphate binding protein | phosphate ABC transporter permease | 0.999 |
| Synpcc7942_2443 | pstB | Synpcc7942_2443 | Synpcc7942_2441 | phosphate ABC transporter permease | phosphate transporter ATP-binding protein; Part of the ABC transporter complex PstSACB involved in phosphate import. Responsible for energy coupling to the transport system (By similarity) | 0.999 |
| Synpcc7942_2442 | pstB | Synpcc7942_2442 | Synpcc7942_2441 | phosphate ABC transporter permease | phosphate transporter ATP-binding protein; Part of the ABC transporter complex PstSACB involved in phosphate import. Responsible for energy coupling to the transport system (By similarity) | 0.999 |
| Synpcc7942_2442 | Synpcc7942_2444 | Synpcc7942_2442 | Synpcc7942_2444 | phosphate ABC transporter permease | phosphate binding protein | 0.999 |
| MgATP | phosphate | 5957 | 1003 | MgATP | Phosphoric acid (also known as orthophosphoric acid or phosphoric(V) acid) is a mineral (inorganic) acid having the chemical formula H3PO4. Orthophosphoric acid molecules can combine with themselves to form a variety of compounds which are also referred to as phosphoric acids, but in a more general way. The term "phosphoric acid" can also refer to a chemical or reagent consisting of phosphoric acids, such as "pyrophosphoric acid" or "triphosphoric acid", but usually orthophosphoric acid.; Orthophosphoric acid refers to "phosphoric acid", which is the IUPAC name for this compound. The prefix "ortho" is used to distinguish the acid from related phosphoric acids, called polyphosphoric acids. Orthophosphoric acid is a non-toxic acid, which, when pure, is a solid at room temperature and pressure. | 0.999 |
| sphX | Synpcc7942_2442 | Synpcc7942_2445 | Synpcc7942_2442 | phosphate binding protein; May be involved in the system for phosphate transport across the cytoplasmic membrane | phosphate ABC transporter permease | 0.998 |
| Synpcc7942_2444 | Synpcc7942_2443 | Synpcc7942_2444 | Synpcc7942_2443 | phosphate binding protein | phosphate ABC transporter permease | 0.998 |
| Synpcc7942_2443 | Synpcc7942_2442 | Synpcc7942_2443 | Synpcc7942_2442 | phosphate ABC transporter permease | phosphate ABC transporter permease | 0.998 |
| Synpcc7942_2443 | Synpcc7942_2444 | Synpcc7942_2443 | Synpcc7942_2444 | phosphate ABC transporter permease | phosphate binding protein | 0.998 |
| Synpcc7942_2442 | sphX | Synpcc7942_2442 | Synpcc7942_2445 | phosphate ABC transporter permease | phosphate binding protein; May be involved in the system for phosphate transport across the cytoplasmic membrane | 0.998 |
| Synpcc7942_2442 | Synpcc7942_2443 | Synpcc7942_2442 | Synpcc7942_2443 | phosphate ABC transporter permease | phosphate ABC transporter permease | 0.998 |
| sphX | pstB | Synpcc7942_2445 | Synpcc7942_2441 | phosphate binding protein; May be involved in the system for phosphate transport across the cytoplasmic membrane | phosphate transporter ATP-binding protein; Part of the ABC transporter complex PstSACB involved in phosphate import. Responsible for energy coupling to the transport system (By similarity) | 0.997 |
| sphX | Synpcc7942_2443 | Synpcc7942_2445 | Synpcc7942_2443 | phosphate binding protein; May be involved in the system for phosphate transport across the cytoplasmic membrane | phosphate ABC transporter permease | 0.997 |
| pstB | sphX | Synpcc7942_2441 | Synpcc7942_2445 | phosphate transporter ATP-binding protein; Part of the ABC transporter complex PstSACB involved in phosphate import. Responsible for energy coupling to the transport system (By similarity) | phosphate binding protein; May be involved in the system for phosphate transport across the cytoplasmic membrane | 0.997 |
| Synpcc7942_2443 | sphX | Synpcc7942_2443 | Synpcc7942_2445 | phosphate ABC transporter permease | phosphate binding protein; May be involved in the system for phosphate transport across the cytoplasmic membrane | 0.997 |
| pstB | Synpcc7942_2444 | Synpcc7942_2441 | Synpcc7942_2444 | phosphate transporter ATP-binding protein; Part of the ABC transporter complex PstSACB involved in phosphate import. Responsible for energy coupling to the transport system (By similarity) | phosphate binding protein | 0.994 |
| Synpcc7942_2444 | pstB | Synpcc7942_2444 | Synpcc7942_2441 | phosphate binding protein | phosphate transporter ATP-binding protein; Part of the ABC transporter complex PstSACB involved in phosphate import. Responsible for energy coupling to the transport system (By similarity) | 0.994 |
| Apigenin-7-glucoside | | | | | | |
| node1 | node2 | node1 accession | node2 accession | node1 annotation | node2 annotation | score |
| rosiglitazone | PPARG | 77998 | ENSP00000287820 | Rosiglitazone (trade name Avandia, GlaxoSmithKline) is an antidiabetic drug in the thiazolidinedione class of drugs. It works as an insulin sensitizer, by binding to the PPAR receptors in fat cells and making the cells more responsive to insulin. It is marketed by the pharmaceutical company GlaxoSmithKline (GSK) as a stand-alone drug or for use in combination with metformin or with glimepiride. First released in 1999, annual sales peaked at approximately $2.5-billion in 2006; however, following a meta-analysis published in the New England Journal of Medicine in 2007 that linked the drug’s use to an increased risk of heart attack, sales plummeted to just $9.5-million in 2012. The drug’s patent expired in 2012. | peroxisome proliferator-activated receptor gamma | 0.999 |
| PPARGC1A | PPARG | ENSP00000264867 | ENSP00000287820 | peroxisome proliferator-activated receptor gamma, coactivator 1 alpha; Transcriptional coactivator for steroid receptors and nuclear receptors. Greatly increases the transcriptional activity of PPARG and thyroid hormone receptor on the uncoupling protein promoter. Can regulate key mitochondrial genes that contribute to the program of adaptive thermogenesis. Plays an essential role in metabolic reprogramming in response to dietary availability through coordination of the expression of a wide array of genes involved in glucose and fatty acid metabolism | peroxisome proliferator-activated receptor gamma | 0.999 |
| PPARG | rosiglitazone | ENSP00000287820 | 77998 | peroxisome proliferator-activated receptor gamma | Rosiglitazone (trade name Avandia, GlaxoSmithKline) is an antidiabetic drug in the thiazolidinedione class of drugs. It works as an insulin sensitizer, by binding to the PPAR receptors in fat cells and making the cells more responsive to insulin. It is marketed by the pharmaceutical company GlaxoSmithKline (GSK) as a stand-alone drug or for use in combination with metformin or with glimepiride. First released in 1999, annual sales peaked at approximately $2.5-billion in 2006; however, following a meta-analysis published in the New England Journal of Medicine in 2007 that linked the drug’s use to an increased risk of heart attack, sales plummeted to just $9.5-million in 2012. The drug’s patent expired in 2012. | 0.999 |
| PPARG | PPARGC1A | ENSP00000287820 | ENSP00000264867 | peroxisome proliferator-activated receptor gamma | peroxisome proliferator-activated receptor gamma, coactivator 1 alpha; Transcriptional coactivator for steroid receptors and nuclear receptors. Greatly increases the transcriptional activity of PPARG and thyroid hormone receptor on the uncoupling protein promoter. Can regulate key mitochondrial genes that contribute to the program of adaptive thermogenesis. Plays an essential role in metabolic reprogramming in response to dietary availability through coordination of the expression of a wide array of genes involved in glucose and fatty acid metabolism | 0.999 |
| pioglitazone | PPARG | 4829 | ENSP00000287820 | Pioglitazone (brand name Actos) is a prescription drug of the class thiazolidinedione (TZD) with hypoglycemic (antihyperglycemic, antidiabetic) action to treat diabetes. Actos was the tenth-best selling drug in the U.S. in 2008, with sales exceeding $2.4 billion. While pioglitazone does decrease blood sugar levels, studies on the main cardiovascular outcomes has not yielded statistically significant results. Its cardiovascular safety profile compares favorably with rosiglitazone (Avandia), which was withdrawn after concerns about an increased risk of cardiac events. Pioglitazone has been found to be associated with bladder tumors and has been withdrawn in some countries. | peroxisome proliferator-activated receptor gamma | 0.998 |
| PPARG | pioglitazone | ENSP00000287820 | 4829 | peroxisome proliferator-activated receptor gamma | Pioglitazone (brand name Actos) is a prescription drug of the class thiazolidinedione (TZD) with hypoglycemic (antihyperglycemic, antidiabetic) action to treat diabetes. Actos was the tenth-best selling drug in the U.S. in 2008, with sales exceeding $2.4 billion. While pioglitazone does decrease blood sugar levels, studies on the main cardiovascular outcomes has not yielded statistically significant results. Its cardiovascular safety profile compares favorably with rosiglitazone (Avandia), which was withdrawn after concerns about an increased risk of cardiac events. Pioglitazone has been found to be associated with bladder tumors and has been withdrawn in some countries. | 0.998 |
| ADIPOR2 | ADIPOQ | ENSP00000349616 | ENSP00000320709 | adiponectin receptor 2; Receptor for globular and full-length adiponectin (APM1), an essential hormone secreted by adipocytes that acts as an antidiabetic. Probably involved in metabolic pathways that regulate lipid metabolism such as fatty acid oxidation. Mediates increased AMPK, PPARA ligand activity, fatty acid oxidation and glucose uptake by adiponectin. Has some intermediate-affinity receptor activity for both globular and full-length adiponectin | adiponectin, C1Q and collagen domain containing; Important adipokine involved in the control of fat metabolism and insulin sensitivity, with direct anti-diabetic, anti-atherogenic and anti-inflammatory activities. Stimulates AMPK phosphorylation and activation in the liver and the skeletal muscle, enhancing glucose utilization and fatty-acid combustion. Antagonizes TNF-alpha by negatively regulating its expression in various tissues such as liver and macrophages, and also by counteracting its effects. Inhibits endothelial NF-kappa-B signaling through a cAMP-dependent pathway. May play [...] | 0.997 |
| ADIPOQ | ADIPOR2 | ENSP00000320709 | ENSP00000349616 | adiponectin, C1Q and collagen domain containing; Important adipokine involved in the control of fat metabolism and insulin sensitivity, with direct anti-diabetic, anti-atherogenic and anti-inflammatory activities. Stimulates AMPK phosphorylation and activation in the liver and the skeletal muscle, enhancing glucose utilization and fatty-acid combustion. Antagonizes TNF-alpha by negatively regulating its expression in various tissues such as liver and macrophages, and also by counteracting its effects. Inhibits endothelial NF-kappa-B signaling through a cAMP-dependent pathway. May play [...] | adiponectin receptor 2; Receptor for globular and full-length adiponectin (APM1), an essential hormone secreted by adipocytes that acts as an antidiabetic. Probably involved in metabolic pathways that regulate lipid metabolism such as fatty acid oxidation. Mediates increased AMPK, PPARA ligand activity, fatty acid oxidation and glucose uptake by adiponectin. Has some intermediate-affinity receptor activity for both globular and full-length adiponectin | 0.997 |
| rosiglitazone | pioglitazone | 77998 | 4829 | Rosiglitazone (trade name Avandia, GlaxoSmithKline) is an antidiabetic drug in the thiazolidinedione class of drugs. It works as an insulin sensitizer, by binding to the PPAR receptors in fat cells and making the cells more responsive to insulin. It is marketed by the pharmaceutical company GlaxoSmithKline (GSK) as a stand-alone drug or for use in combination with metformin or with glimepiride. First released in 1999, annual sales peaked at approximately $2.5-billion in 2006; however, following a meta-analysis published in the New England Journal of Medicine in 2007 that linked the drug’s use to an increased risk of heart attack, sales plummeted to just $9.5-million in 2012. The drug’s patent expired in 2012. | Pioglitazone (brand name Actos) is a prescription drug of the class thiazolidinedione (TZD) with hypoglycemic (antihyperglycemic, antidiabetic) action to treat diabetes. Actos was the tenth-best selling drug in the U.S. in 2008, with sales exceeding $2.4 billion. While pioglitazone does decrease blood sugar levels, studies on the main cardiovascular outcomes has not yielded statistically significant results. Its cardiovascular safety profile compares favorably with rosiglitazone (Avandia), which was withdrawn after concerns about an increased risk of cardiac events. Pioglitazone has been found to be associated with bladder tumors and has been withdrawn in some countries. | 0.996 |
| pioglitazone | rosiglitazone | 4829 | 77998 | Pioglitazone (brand name Actos) is a prescription drug of the class thiazolidinedione (TZD) with hypoglycemic (antihyperglycemic, antidiabetic) action to treat diabetes. Actos was the tenth-best selling drug in the U.S. in 2008, with sales exceeding $2.4 billion. While pioglitazone does decrease blood sugar levels, studies on the main cardiovascular outcomes has not yielded statistically significant results. Its cardiovascular safety profile compares favorably with rosiglitazone (Avandia), which was withdrawn after concerns about an increased risk of cardiac events. Pioglitazone has been found to be associated with bladder tumors and has been withdrawn in some countries. | Rosiglitazone (trade name Avandia, GlaxoSmithKline) is an antidiabetic drug in the thiazolidinedione class of drugs. It works as an insulin sensitizer, by binding to the PPAR receptors in fat cells and making the cells more responsive to insulin. It is marketed by the pharmaceutical company GlaxoSmithKline (GSK) as a stand-alone drug or for use in combination with metformin or with glimepiride. First released in 1999, annual sales peaked at approximately $2.5-billion in 2006; however, following a meta-analysis published in the New England Journal of Medicine in 2007 that linked the drug’s use to an increased risk of heart attack, sales plummeted to just $9.5-million in 2012. The drug’s patent expired in 2012. | 0.996 |
| TBC1D4 | SLC2A4 | ENSP00000366863 | ENSP00000320935 | TBC1 domain family, member 4; May act as a GTPase-activating protein for RAB2A, RAB8A, RAB10 and RAB14. Isoform 2 promotes insulin-induced glucose transporter SLC2A4/GLUT4 translocation at the plasma membrane, thus increasing glucose uptake | solute carrier family 2 (facilitated glucose transporter), member 4; Insulin-regulated facilitative glucose transporter | 0.996 |
| SLC2A4 | MYO1C | ENSP00000320935 | ENSP00000352834 | solute carrier family 2 (facilitated glucose transporter), member 4; Insulin-regulated facilitative glucose transporter | myosin IC; Myosins are actin-based motor molecules with ATPase activity. Unconventional myosins serve in intracellular movements. Their highly divergent tails are presumed to bind to membranous compartments, which would be moved relative to actin filaments. Involved in glucose transporter recycling in response to insulin by regulating movement of intracellular GLUT4-containing vesicles to the plasma membrane. Component of the hair cell’s (the sensory cells of the inner ear) adaptation-motor complex. Acts as a mediator of adaptation of mechanoelectrical transduction in stereocilia of ve [...] | 0.996 |
| SLC2A4 | TBC1D4 | ENSP00000320935 | ENSP00000366863 | solute carrier family 2 (facilitated glucose transporter), member 4; Insulin-regulated facilitative glucose transporter | TBC1 domain family, member 4; May act as a GTPase-activating protein for RAB2A, RAB8A, RAB10 and RAB14. Isoform 2 promotes insulin-induced glucose transporter SLC2A4/GLUT4 translocation at the plasma membrane, thus increasing glucose uptake | 0.996 |
| MYO1C | SLC2A4 | ENSP00000352834 | ENSP00000320935 | myosin IC; Myosins are actin-based motor molecules with ATPase activity. Unconventional myosins serve in intracellular movements. Their highly divergent tails are presumed to bind to membranous compartments, which would be moved relative to actin filaments. Involved in glucose transporter recycling in response to insulin by regulating movement of intracellular GLUT4-containing vesicles to the plasma membrane. Component of the hair cell’s (the sensory cells of the inner ear) adaptation-motor complex. Acts as a mediator of adaptation of mechanoelectrical transduction in stereocilia of ve [...] | solute carrier family 2 (facilitated glucose transporter), member 4; Insulin-regulated facilitative glucose transporter | 0.996 |
| PPARG | ADIPOQ | ENSP00000287820 | ENSP00000320709 | peroxisome proliferator-activated receptor gamma | adiponectin, C1Q and collagen domain containing; Important adipokine involved in the control of fat metabolism and insulin sensitivity, with direct anti-diabetic, anti-atherogenic and anti-inflammatory activities. Stimulates AMPK phosphorylation and activation in the liver and the skeletal muscle, enhancing glucose utilization and fatty-acid combustion. Antagonizes TNF-alpha by negatively regulating its expression in various tissues such as liver and macrophages, and also by counteracting its effects. Inhibits endothelial NF-kappa-B signaling through a cAMP-dependent pathway. May play [...] | 0.995 |
| ADIPOQ | PPARG | ENSP00000320709 | ENSP00000287820 | adiponectin, C1Q and collagen domain containing; Important adipokine involved in the control of fat metabolism and insulin sensitivity, with direct anti-diabetic, anti-atherogenic and anti-inflammatory activities. Stimulates AMPK phosphorylation and activation in the liver and the skeletal muscle, enhancing glucose utilization and fatty-acid combustion. Antagonizes TNF-alpha by negatively regulating its expression in various tissues such as liver and macrophages, and also by counteracting its effects. Inhibits endothelial NF-kappa-B signaling through a cAMP-dependent pathway. May play [...] | peroxisome proliferator-activated receptor gamma | 0.995 |
| SLC2A4 | PPARG | ENSP00000320935 | ENSP00000287820 | solute carrier family 2 (facilitated glucose transporter), member 4; Insulin-regulated facilitative glucose transporter | peroxisome proliferator-activated receptor gamma | 0.994 |
| PPARG | SLC2A4 | ENSP00000287820 | ENSP00000320935 | peroxisome proliferator-activated receptor gamma | solute carrier family 2 (facilitated glucose transporter), member 4; Insulin-regulated facilitative glucose transporter | 0.994 |
| ADIPOR1 | ADIPOQ | ENSP00000341785 | ENSP00000320709 | adiponectin receptor 1; Receptor for globular and full-length adiponectin (APM1), an essential hormone secreted by adipocytes that acts as an antidiabetic. Probably involved in metabolic pathways that regulate lipid metabolism such as fatty acid oxidation. Mediates increased AMPK, PPARA ligand activity, fatty acid oxidation and glucose uptake by adiponectin. Has some high-affinity receptor for globular adiponectin but low-affinity receptor for full-length adiponectin | adiponectin, C1Q and collagen domain containing; Important adipokine involved in the control of fat metabolism and insulin sensitivity, with direct anti-diabetic, anti-atherogenic and anti-inflammatory activities. Stimulates AMPK phosphorylation and activation in the liver and the skeletal muscle, enhancing glucose utilization and fatty-acid combustion. Antagonizes TNF-alpha by negatively regulating its expression in various tissues such as liver and macrophages, and also by counteracting its effects. Inhibits endothelial NF-kappa-B signaling through a cAMP-dependent pathway. May play [...] | 0.994 |
| ADIPOQ | ADIPOR1 | ENSP00000320709 | ENSP00000341785 | adiponectin, C1Q and collagen domain containing; Important adipokine involved in the control of fat metabolism and insulin sensitivity, with direct anti-diabetic, anti-atherogenic and anti-inflammatory activities. Stimulates AMPK phosphorylation and activation in the liver and the skeletal muscle, enhancing glucose utilization and fatty-acid combustion. Antagonizes TNF-alpha by negatively regulating its expression in various tissues such as liver and macrophages, and also by counteracting its effects. Inhibits endothelial NF-kappa-B signaling through a cAMP-dependent pathway. May play [...] | adiponectin receptor 1; Receptor for globular and full-length adiponectin (APM1), an essential hormone secreted by adipocytes that acts as an antidiabetic. Probably involved in metabolic pathways that regulate lipid metabolism such as fatty acid oxidation. Mediates increased AMPK, PPARA ligand activity, fatty acid oxidation and glucose uptake by adiponectin. Has some high-affinity receptor for globular adiponectin but low-affinity receptor for full-length adiponectin | 0.994 |
| cosmosiin | ADIPOQ | 5280704 | ENSP00000320709 | Apigetrin is a chemical compound that can be found in dandelion coffee and in "Teucrium gnaphalodes". | adiponectin, C1Q and collagen domain containing; Important adipokine involved in the control of fat metabolism and insulin sensitivity, with direct anti-diabetic, anti-atherogenic and anti-inflammatory activities. Stimulates AMPK phosphorylation and activation in the liver and the skeletal muscle, enhancing glucose utilization and fatty-acid combustion. Antagonizes TNF-alpha by negatively regulating its expression in various tissues such as liver and macrophages, and also by counteracting its effects. Inhibits endothelial NF-kappa-B signaling through a cAMP-dependent pathway. May play [...] | 0.800 |
| ADIPOQ | cosmosiin | ENSP00000320709 | 5280704 | adiponectin, C1Q and collagen domain containing; Important adipokine involved in the control of fat metabolism and insulin sensitivity, with direct anti-diabetic, anti-atherogenic and anti-inflammatory activities. Stimulates AMPK phosphorylation and activation in the liver and the skeletal muscle, enhancing glucose utilization and fatty-acid combustion. Antagonizes TNF-alpha by negatively regulating its expression in various tissues such as liver and macrophages, and also by counteracting its effects. Inhibits endothelial NF-kappa-B signaling through a cAMP-dependent pathway. May play [...] | Apigetrin is a chemical compound that can be found in dandelion coffee and in "Teucrium gnaphalodes". | 0.800 |
| SLC2A4 | ADIPOQ | ENSP00000320935 | ENSP00000320709 | solute carrier family 2 (facilitated glucose transporter), member 4; Insulin-regulated facilitative glucose transporter | adiponectin, C1Q and collagen domain containing; Important adipokine involved in the control of fat metabolism and insulin sensitivity, with direct anti-diabetic, anti-atherogenic and anti-inflammatory activities. Stimulates AMPK phosphorylation and activation in the liver and the skeletal muscle, enhancing glucose utilization and fatty-acid combustion. Antagonizes TNF-alpha by negatively regulating its expression in various tissues such as liver and macrophages, and also by counteracting its effects. Inhibits endothelial NF-kappa-B signaling through a cAMP-dependent pathway. May play [...] | 0.700 |
| ADIPOQ | SLC2A4 | ENSP00000320709 | ENSP00000320935 | adiponectin, C1Q and collagen domain containing; Important adipokine involved in the control of fat metabolism and insulin sensitivity, with direct anti-diabetic, anti-atherogenic and anti-inflammatory activities. Stimulates AMPK phosphorylation and activation in the liver and the skeletal muscle, enhancing glucose utilization and fatty-acid combustion. Antagonizes TNF-alpha by negatively regulating its expression in various tissues such as liver and macrophages, and also by counteracting its effects. Inhibits endothelial NF-kappa-B signaling through a cAMP-dependent pathway. May play [...] | solute carrier family 2 (facilitated glucose transporter), member 4; Insulin-regulated facilitative glucose transporter | 0.700 |
| PPARG | ADIPOR1 | ENSP00000287820 | ENSP00000341785 | peroxisome proliferator-activated receptor gamma | adiponectin receptor 1; Receptor for globular and full-length adiponectin (APM1), an essential hormone secreted by adipocytes that acts as an antidiabetic. Probably involved in metabolic pathways that regulate lipid metabolism such as fatty acid oxidation. Mediates increased AMPK, PPARA ligand activity, fatty acid oxidation and glucose uptake by adiponectin. Has some high-affinity receptor for globular adiponectin but low-affinity receptor for full-length adiponectin | 0.581 |
| ADIPOR1 | PPARG | ENSP00000341785 | ENSP00000287820 | adiponectin receptor 1; Receptor for globular and full-length adiponectin (APM1), an essential hormone secreted by adipocytes that acts as an antidiabetic. Probably involved in metabolic pathways that regulate lipid metabolism such as fatty acid oxidation. Mediates increased AMPK, PPARA ligand activity, fatty acid oxidation and glucose uptake by adiponectin. Has some high-affinity receptor for globular adiponectin but low-affinity receptor for full-length adiponectin | peroxisome proliferator-activated receptor gamma | 0.581 |
| SLC2A4 | ADIPOR1 | ENSP00000320935 | ENSP00000341785 | solute carrier family 2 (facilitated glucose transporter), member 4; Insulin-regulated facilitative glucose transporter | adiponectin receptor 1; Receptor for globular and full-length adiponectin (APM1), an essential hormone secreted by adipocytes that acts as an antidiabetic. Probably involved in metabolic pathways that regulate lipid metabolism such as fatty acid oxidation. Mediates increased AMPK, PPARA ligand activity, fatty acid oxidation and glucose uptake by adiponectin. Has some high-affinity receptor for globular adiponectin but low-affinity receptor for full-length adiponectin | 0.571 |
| ADIPOR1 | SLC2A4 | ENSP00000341785 | ENSP00000320935 | adiponectin receptor 1; Receptor for globular and full-length adiponectin (APM1), an essential hormone secreted by adipocytes that acts as an antidiabetic. Probably involved in metabolic pathways that regulate lipid metabolism such as fatty acid oxidation. Mediates increased AMPK, PPARA ligand activity, fatty acid oxidation and glucose uptake by adiponectin. Has some high-affinity receptor for globular adiponectin but low-affinity receptor for full-length adiponectin | solute carrier family 2 (facilitated glucose transporter), member 4; Insulin-regulated facilitative glucose transporter | 0.571 |
| SLC2A4 | ADIPOR2 | ENSP00000320935 | ENSP00000349616 | solute carrier family 2 (facilitated glucose transporter), member 4; Insulin-regulated facilitative glucose transporter | adiponectin receptor 2; Receptor for globular and full-length adiponectin (APM1), an essential hormone secreted by adipocytes that acts as an antidiabetic. Probably involved in metabolic pathways that regulate lipid metabolism such as fatty acid oxidation. Mediates increased AMPK, PPARA ligand activity, fatty acid oxidation and glucose uptake by adiponectin. Has some intermediate-affinity receptor activity for both globular and full-length adiponectin | 0.542 |
| ADIPOR2 | SLC2A4 | ENSP00000349616 | ENSP00000320935 | adiponectin receptor 2; Receptor for globular and full-length adiponectin (APM1), an essential hormone secreted by adipocytes that acts as an antidiabetic. Probably involved in metabolic pathways that regulate lipid metabolism such as fatty acid oxidation. Mediates increased AMPK, PPARA ligand activity, fatty acid oxidation and glucose uptake by adiponectin. Has some intermediate-affinity receptor activity for both globular and full-length adiponectin | solute carrier family 2 (facilitated glucose transporter), member 4; Insulin-regulated facilitative glucose transporter | 0.542 |
| TBC1D4 | MYO1C | ENSP00000366863 | ENSP00000352834 | TBC1 domain family, member 4; May act as a GTPase-activating protein for RAB2A, RAB8A, RAB10 and RAB14. Isoform 2 promotes insulin-induced glucose transporter SLC2A4/GLUT4 translocation at the plasma membrane, thus increasing glucose uptake | myosin IC; Myosins are actin-based motor molecules with ATPase activity. Unconventional myosins serve in intracellular movements. Their highly divergent tails are presumed to bind to membranous compartments, which would be moved relative to actin filaments. Involved in glucose transporter recycling in response to insulin by regulating movement of intracellular GLUT4-containing vesicles to the plasma membrane. Component of the hair cell’s (the sensory cells of the inner ear) adaptation-motor complex. Acts as a mediator of adaptation of mechanoelectrical transduction in stereocilia of ve [...] | 0.407 |
| MYO1C | TBC1D4 | ENSP00000352834 | ENSP00000366863 | myosin IC; Myosins are actin-based motor molecules with ATPase activity. Unconventional myosins serve in intracellular movements. Their highly divergent tails are presumed to bind to membranous compartments, which would be moved relative to actin filaments. Involved in glucose transporter recycling in response to insulin by regulating movement of intracellular GLUT4-containing vesicles to the plasma membrane. Component of the hair cell’s (the sensory cells of the inner ear) adaptation-motor complex. Acts as a mediator of adaptation of mechanoelectrical transduction in stereocilia of ve [...] | TBC1 domain family, member 4; May act as a GTPase-activating protein for RAB2A, RAB8A, RAB10 and RAB14. Isoform 2 promotes insulin-induced glucose transporter SLC2A4/GLUT4 translocation at the plasma membrane, thus increasing glucose uptake | 0.407 |

**Table S4.** Antioxidant activity of the prepared nanoparticles

| Sample | IC_50_ mean ± SD (µg/ml) |
| --- | --- |
| Juniperus phoenicea L. Extract | 1542.77 ± 44.5158 |
| JP-NLCs | 1491.41 ± 51.4179 |

**Table S5.** Percentage of inhibitory effect of the prepared nanoparticles against lung cancer cell line

| Sample conc. (µg/ml) | Fluorouracil | | | Juniperus phoenicea L. extract | | | JP-NLCs | | | Combined Fluorouracil and JP-NLCs | | |
| --- | --- | --- | --- | --- | --- | --- | --- | --- | --- | --- | --- | --- |
|  | Inhibitory % | S.D. (±) | Inhibitory % | | S.D. (±) | Inhibitory % | | S.D. (±) | Inhibitory % | | S.D. (±) |  |
| 500 | 95.21 | 0.37 | 91.41 | | 0.37 | 98.57 | | 0.09 | 98.91 | | 0.13 |  |
| 250 | 86.59 | 0.65 | 82.58 | | 1.66 | 94.94 | | 0.12 | 96.53 | | 0.19 |  |
| 125 | 75.41 | 1.23 | 68.22 | | 2.94 | 88.32 | | 0.14 | 90.32 | | 0.26 |  |
| 62.5 | 53.88 | 1.46 | 43.59 | | 2.37 | 79.69 | | 0.53 | 83.57 | | 0.31 |  |
| 31.25 | 26.25 | 1.09 | 21.8 | | 1.42 | 73.52 | | 1.46 | 75.22 | | 0.84 |  |
| 15.6 | 13.54 | 0.82 | 7.64 | | 0.62 | 65.31 | | 1.78 | 69.46 | | 0.92 |  |
| 7.8 | 6.92 | 0.61 | 0.55 | | 0.39 | 47.07 | | 2.09 | 54.83 | | 1.05 |  |
| 3.9 | 1.09 | 0.35 | 0 | | - | 36.82 | | 1.64 | 43.59 | | 1.33 |  |
| 2 | 0 | - | 0 | | - | 29.36 | | 2.08 | 32.61 | | 0.67 |  |
| 1 | 0 | - | 0 | | - | 18.51 | | 1.75 | 25.06 | | 0.82 |  |
| 0 | 0 | - | 0 | | - | 0 | | - | 0 | | - |  |

**Table** S**6.** The CI and DRI values of the formulated combination

| **Total Dose (Units)** | **Effect (ƒ_a_)** | **CI Value** | **Interaction Type** | **DRI (Drug JP-NLCs)** | **DRI (Drug Fluorouracil)** |
| --- | --- | --- | --- | --- | --- |
| **1100.0** | 0.12 | 1.22748 | Antagonism | 9.76152 | 0.88886 |
| **550.0** | 0.25 | 1.08967 | Antagonism | 9.02492 | 1.02159 |
| **275.0** | 0.42 | 0.89987 | Synergism | 9.23850 | 1.26322 |
| **137.5** | 0.60 | 0.72308 | Synergism | 9.85384 | 1.60877 |
| **68.75** | **0.76** | **0.59057** | **Strong Synergy** | **10.3394** | **2.02489** |
| **34.375** | 0.96 | 1.14427 | Antagonism | 3.59904 | 1.15418 |
